# Multilevel Meta‐Analysis of Treatment Options for Patients With Iliopsoas Impingement Syndrome After Total Hip Arthroplasty

**DOI:** 10.1111/os.70021

**Published:** 2025-05-23

**Authors:** Nikolai Ramadanov, Maximilian Voss, Robert Hable, Robert Prill, Dobromir Dimitrov, Marco Ezechieli, Ingo J. Banke, Roland Becker

**Affiliations:** ^1^ Center of Orthopaedics and Traumatology Brandenburg Medical School, University Hospital Brandenburg an der Havel Brandenburg an der Havel Germany; ^2^ Faculty of Health Science Brandenburg, Brandenburg Medical School Theodor Fontane Brandenburg an der Havel Germany; ^3^ Faculty of Applied Computer Science Deggendorf Institute of Technology Deggendorf Germany; ^4^ Department of Surgical Diseases Faculty of Medicine, Medical University of Pleven Pleven Bulgaria; ^5^ Center of Orthopaedics and Traumatology Vincenz Hospital Paderborn Paderborn Germany; ^6^ Clinic of Orthopedics and Sports Orthopedics Klinikum Rechts der Isar, Technical University of Munich Munich Germany

**Keywords:** acetabular cup revision, conservative management, endoscopic tenotomy, iliopsoas impingement, meta‐analysis, total hip arthroplasty

## Abstract

**Objective:**

Iliopsoas impingement (IPI) syndrome is a significant complication following total hip arthroplasty (THA), often leading to pain and reduced hip function. Despite its clinical relevance, the optimal treatment strategy remains unclear, with varying success rates reported across different interventions. This study aims to compare four treatment options (endoscopic, acetabular cup revision, open tenotomy and conservative management) for patients with IPI syndrome after THA by comparing outcomes in terms of function, pain, complications, and reoperations through a multilevel meta‐analysis.

**Methods:**

A literature search was conducted in the following databases until 30 November 2024: PubMed, CENTRAL, Epistemonikos, and Embase. A frequentist multilevel meta‐analysis was performed using a random effects model with an inverse variance and restricted maximum likelihood heterogeneity estimator with Hartung‐Knapp adjustment. Means with 95% confidence intervals (CIs) were calculated separately in the four treatment groups. Then, a test for subgroup differences in multilevel meta‐analysis was performed to determine whether there is a statistically significant difference between the means of the four groups.

**Results:**

The systematic review included 15 studies with 425 patients. The test for subgroup differences showed no statistically significant difference between the four treatment subgroups in Harris Hip Score (HHS) post‐intervention (*F* = 2.0; *df* = 3, 7; *p* = 0.20), in HHS difference (*F* = 2.0; *df* = 3, 6; *p* = 0.22), and in functional minimal clinically important differences (MCID) post‐intervention (*F* = 1.0; *df* = 3, 2; *p* = 0.42). The conservative management group exhibited the lowest mean HHS (70.3 points).

**Conclusions:**

Surgical interventions, including endoscopic tenotomy, acetabular cup revision, and open tenotomy, are effective in achieving meaningful functional improvements in IPI patients. While conservative management was the least effective of all treatment groups, the differences did not reach statistical significance.

## Graphical abstract

A multilevel meta‐analysis (15 studies, 425 patients) evaluated four treatments—endoscopic tenotomy, acetabular cup revision, open tenotomy, and conservative management—for iliopsoas impingement (IPI) post‐total hip arthroplasty (THA). Outcomes assessed included function, pain, complications, and reoperations. Subgroup differences in post‐treatment Harris Hip Scores (HHS) and functional improvements were analyzed. Surgical methods achieved better functional outcomes than conservative management, which had the lowest mean HHS (70.3). However, subgroup differences were not statistically significant. Surgical interventions improve functionality in IPI patients. Conservative management is less effective, but differences between treatments are not significant.

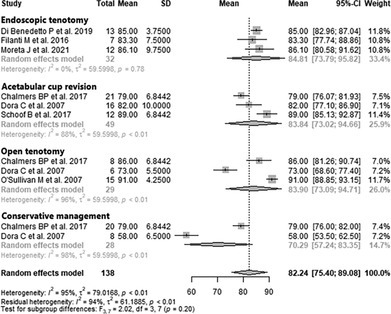

**Abbreviations**
CENTRAL: Central Register of Controlled Trials
GRADE: Grading of Recommendations, Assessment, Development, and Evaluation
HHS: Harris Hip Score
iHOT: International Hip Outcome Tool
IPI: iliopsoas impingement
MCID: minimal clinically important difference
mHHS: modified Harris Hip Score
OHS: Oxford Hip Score
PRISMA: Preferred Reporting Items for Systematic Reviews and Meta‐Analyses
PROM: patient‐reported outcome measure
PROSPERO: International Prospective Register of Systematic Reviews
SANE: Single Assessment Numeric Evaluation
THA: total hip arthroplasty

## 1 Introduction

Iliopsoas impingement (IPI) syndrome is a well‐recognized complication following total hip arthroplasty (THA), often presenting as persistent pain and reduced function, particularly during activities involving hip flexion [1, 2]. This syndrome has gained attention due to its significant impact on patient satisfaction and outcomes after THA. Historically, the etiology behind post‐THA discomfort remained elusive, especially in cases with no visible structural problems. However, recent studies suggest that IPI may be a contributing factor, particularly concerning acetabular cup positioning [3, 4]. Although the incidence of IPI syndrome after THA is relatively low, its significant impact on patient quality of life and association with modifiable factors such as acetabular cup positioning warrants further investigation into optimal management strategies.

Cup orientation, including inclination, anteversion, and prominence, influences the likelihood of iliopsoas irritation. In his 1978 study, Lewinnek et al. suggested a “safe zone” for acetabular component orientation in THA with an ideal inclination angle of 40° ± 10° and an anteversion angle of 15° ± 10° [5]. However, even within this safe zone, iliopsoas irritation can occur in rare cases, highlighting the multifactorial nature of this condition. Currently, there is no uniform recommendation for the optimal treatment approach, and management strategies often depend on individual patient factors and physician preferences. Conservative treatments, such as physiotherapy and anti‐inflammatory medications, often provide only moderate relief, with studies indicating that pain resolves in about half of the cases [6]. Surgical options, including endoscopic or open tenotomy, have shown better results, with many patients experiencing significant pain relief and improved hip function. Satisfaction rates with these procedures are generally high though some individuals may develop hip flexor weakness, potentially affecting daily activities [7]. Acetabular cup revision can correct implant‐related impingement but is a highly invasive procedure with considerable risks, such as infection and loosening [8].

A systematic review is a structured, comprehensive analysis of existing research studies on a specific topic, using rigorous methods to identify, evaluate, and synthesize findings to provide a high‐level overview of current evidence [9]. Currently, only four systematic reviews address treatment options for managing IPI following THA [10, 11, 12, 13]. Although these studies provide insights into surgical techniques and management approaches, they have some severe limitations. Notably, only one systematic review [13] performed a meta‐analysis, which lacked an adequate sample size of primary studies and patients, leading to inconclusive results. A more methodologically rigorous meta‐analysis with a larger dataset is needed to establish reliable evidence on the efficacy of various treatment options for IPI after THA.

The present study aims to evaluate four treatment options (endoscopic, acetabular cup revision, open tenotomy and conservative management) for patients with IPI after THA by comparing outcomes in terms of function, pain, complications, and reoperations through a multilevel meta‐analysis.

## 2 Methods

### 2.1 Reporting Guidelines and Protocol Registration

The study protocol was registered in the International Prospective Register of Systematic Reviews (PROSPERO) on 06 November 2024 (CRD42024606752). The updated PRISMA (Preferred Reporting Items for Systematic Reviews and Meta‐Analyses) guidelines [14] were strictly followed. The PRISMA checklist is available in the Supporting Information (Table S1).

### 2.2 Data Sources and Search Strategies

A comprehensive literature search was conducted in the following databases until 30 November 2024: PubMed, the Cochrane Library's Central Register of Controlled Trials (CENTRAL), Epistemonikos, and Embase. A Boolean search strategy was used to identify studies that address treatment options for IPI patients. The search strategy was tailored to each database's syntax: (((iliopsoas impingement) OR (IPI)) AND ((arthroscopy) OR (endoscopy) OR (arthroscopic) OR (endoscopic) OR (conservative) OR (tenotomy))).

### 2.3 Study Screening and Selection

In a stepwise selection process, two independent reviewers (NR and MV) first assessed the titles and abstracts of the records in the databases. Then, the full texts were retrieved and re‐assessed by both reviewers (NR and MV) to determine inclusion. The final decision to include each study was made by consensus between the two reviewers (NR and MV). Inter‐reviewer agreement was measured using the kappa coefficient (κ). Discrepancies between the two reviewers were resolved through in‐depth discussion until a consensus was reached, ensuring consistency in study selection and data extraction.

### 2.4 Inclusion and Exclusion Criteria

The inclusion criteria for this review considered all types of primary prospective and retrospective studies, including case reports, focusing on the treatment of IPI. Gray literature was excluded to ensure the inclusion of studies with rigorous peer review and consistent reporting standards, thereby minimizing potential sources of bias. Four main categories of interventions were considered: endoscopic or open tenotomy, acetabular cup revision, and conservative treatment. Conservative treatment included non‐surgical approaches such as physiotherapy, pharmacological pain relief, and lifestyle changes. Primary studies that did not report outcomes relevant to the research question were excluded. Primary studies with unclear or incomplete data were also excluded.

### 2.5 Types of Outcome Measures

Hip function and quality of life were measured using the following patient‐reported outcome measures (PROMs):

Harris Hip Score (HHS) and modified HHS (mHHS) to assess hip function and pain on a scale from 0 (worst) to 100 (best).

The HHS difference [15] is the difference between the reported HHS pre‐intervention and the measured HHS post‐intervention. It was obtained by arithmetically subtracting the two values.

International Hip Outcome Tool (iHOT) for assessing hip‐related quality of life on a scale from 0 (worst) to 100 (best).

Oxford Hip Score (OHS), assessing function and pain with 12 items on a scale from 0 (worst) to 48 (best).

Single Assessment Numeric Evaluation (SANE) hip, a single‐item measure where patients rate their hip function on a scale from 0 (worst) to 100 (best).

### 2.6 Data Extraction and Analysis

Data extraction was performed by two independent reviewers (NR and MV). Disagreements were resolved by discussion and consensus. Key study details, including the first author, year of publication, study origin, number of patients included, patient characteristics, study design, risk of bias, relevant outcome parameters, and duration of follow‐up were extracted. Conversion to minimal clinically important difference (MCID) [16] units was performed by dividing the PROM score by the most conservative MCID reported in the literature [17, 18, 19, 20, 21] (Table 1). In primary studies where multiple PROMs were reported at different time points, we included the PROM of the latest follow‐up. The data extraction set is available in the Supporting Information (Table S2).

**TABLE 1:** Minimal clinically important difference (MCID) units used for each patient‐reported outcome measure (PROM).

PROM	MCID unit
HHS	7.90 [17]
mHHS	8.20 [18]
iHOT‐12	12.90 [19]
OHS	11.00 [20]
SANE	7.00 [21]

Abbreviations: HHS, Harris Hip Score; iHOT, International Hip Outcome Tool; MCID, Minimal clinically important difference; mHHS, Modified Harris Hip Score; OHS, Oxford Hip Score; PROM, Patient‐reported outcome measure; SANE, Single Assessment Numeric Evaluation.

### 2.7 Quality Assessment

Two independent reviewers (NR and MV) individually assessed the quality of the primary studies included. Primary studies were assessed using the Risk Of Bias In Non‐randomized Studies of Interventions (ROBINS‐I) tool [22] for non‐randomized studies. The level of evidence for each outcome parameter was determined using the Grading of Recommendations, Assessment, Development, and Evaluation (GRADE) tool [23]. Disagreements between the two reviewers (NR and MV) were resolved by discussion. The ROBINS‐I tool was used to assess the risk of bias, focusing on confounding, selection bias, and deviations from intended interventions. The GRADE criteria emphasized study design, risk of bias, inconsistency, indirectness, and imprecision to determine the overall quality of evidence for each outcome parameter.

### 2.8 Measures of Treatment Effect

To comprehensively assess the treatment effects between conservative treatment, endoscopic or open tenotomy, and acetabular cup revision, a frequentist multilevel meta‐analysis was performed using a random effects model with inverse variance and restricted maximum likelihood heterogeneity estimator with Hartung‐Knapp adjustment. The assessed functional scores are continuous variables for which means with 95% confidence intervals (CIs) were calculated separately in the four treatment subgroups. Then, a test for subgroup differences in multilevel meta‐analysis was performed to determine whether there is a statistically significant difference between the means of the four subgroups. Statistical heterogeneity was assessed using the Higgins *I* ^2^ test, which categorizes heterogeneity as low (< 25%), moderate (25%–75%), or high (> 75%). The results of the multilevel meta‐analysis were presented in forest plots. Additionally, a meta‐regression analysis was performed to examine potential sources of heterogeneity, such as patient demographic parameters. A level of *p* = 0.05 was considered statistically significant. All statistical calculations were performed by an experienced statistician (RH) using the R packages meta and metafor.

## 3 Results

### 3.1 Systematic Review

**FIGURE 1:**
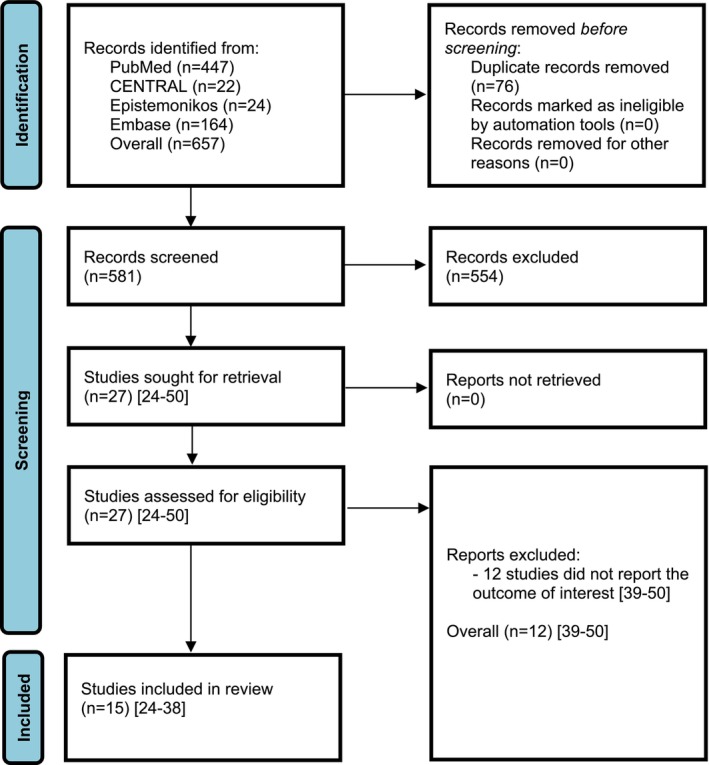
PRISMA flow diagram of the search results and selection according to our inclusion criteria. CENTRAL: Cochrane Central Register of Controlled Trials.

After removing 76 duplicate records, 581 records were screened for titles and abstracts with high inter‐reviewer agreement (*κ* = 0.98). A total of 27 studies [24, 25, 26, 27, 28, 29, 30, 31, 32, 33, 34, 35, 36, 37, 38, 39, 40, 41, 42, 43, 44, 45, 46, 47, 48, 49, 50] were assessed for eligibility with full inter‐reviewer agreement (*κ* = 1.0). Twelve studies were excluded because they did not report relevant outcomes [39, 40, 41, 42, 43, 44, 45, 46, 47, 48, 49, 50]. The systematic review finally included 15 studies [24, 25, 26, 27, 28, 29, 30, 31, 32, 33, 34, 35, 36, 37, 38] with 425 patients that met the eligibility criteria for the multi‐level meta‐analysis (Figure 1).

### 3.2 Characteristics of Included Studies

Table 2 shows the main characteristics of the 15 studies included in the review (Table 2). A total of 273 patients were included in the endoscopic tenotomy group, 95 in the revision group, 29 in the open tenotomy group, and 28 in the conservative management group. Ten studies [25, 27, 29, 30, 31, 32, 33, 36, 37, 38] focused on endoscopic tenotomy, four [24, 26, 28, 35] on acetabular cup revision, three [26, 28, 34] on open tenotomy, and two [26, 28] on conservative management. Further details of the patient cohort are shown in Table 2.

**TABLE 2:** Main characteristics of the studies and the patient cohort.

Author	Year of publication	Origin	Study design	Procedure	Patients *N*	Male sex *N* (%)	Age years ± SD (range)	BMI kg/m^2^ ± SD (range)	Inclination degrees ± SD (range)	Prominence mm ± SD (range)	Anteversion degrees ± SD (range)	HHS preoperative points ± SD (range)	mHHS preoperative points ± SD (range)	Recurrence *N*	Revision *N*	Complications *N*
Batailler et al. [24]	2017	France	Retrospective case series	Revision	46	13 (28)	66.0 ± 12.0 (44.0–85.0)	27.5 ± 4.8 (18.0–45.0)	50.0 ± 10.0 (29.0–75.0)	9.9 ± 4.5 (2.0–22.0)	8.0 ± 9.0 (−10.0–35.0)	NR	NR	NR	NR	NR
Bonano et al. [25]	2023	USA	Retrospective case series	Endoscopic	24	8 (33)	69.0 (6205–74.3)	30.4	45.0 (37.8–49.0)	7.0 (2.3–12.0)	30.0 (24.5–38.0)	NR	57.0 (43.0–60.0)	1	1	2
Chalmers et al. [26]	2017	USA	Retrospective cohort study	Revision	21	16 (55)	62.0	NR	NR	8.0 (3.0–18.0)	NR	58.0	NR	NR	NR	NR
				Open tenotomy	8	0 (0)	62.0	NR	NR	8.0 (3.0–18.0)	NR	53.0	NR	NR	NR	NR
				Conservative management	20	7 (35)	69.0	NR	NR	8.0 (2.0–18.0)	NR	NR	NR	NR	NR	NR
Di Benedetto et al. [27]	2019	Italy	Retrospective case series	Endoscopic	13	9 (62)	65.0 (47.0–82.0)	NR	NR	NR	NR	668 (48.9–81.8)	NR	0	0	0
Dora et al. [28]	2007	Switzerland	Retrospective cohort study	Revision	16	6 (38)	62.0 (29.0–82.0)	NR	NR	NR	NR	60.0 (48.0–85.0)	NR	NR	NR	NR
				Open tenotomy	6	3 (50)	73.0 (67.0–81.0)	NR	NR	NR	NR	59.0 (56.0–67.0)	NR	NR	NR	NR
				Conservative management	8	3 (38)	57.0 (37.0–78.0)	NR	NR	NR	NR	57.0 (50.0–68.0)	NR	NR	NR	NR
Filanti et al. [29]	2016	Italy	Retrospective case series	Endoscopic	7	4 (57)	57.0 (29.0–77.0)	NR	NR	NR	NR	44.1 (32.0–56.0)	NR	1	0	0
Finsterwald et al. [30]	2024	Australia	Retrospective case series	Endoscopic	36	11 (31)	62.0 ±12.0 (27.0–83.0)	28.9 ± 4.3 (23.2–38.5)	42.5 ± 7.2 (30.0–59.0)	NR	18.3 ±9.5 (− 1.0–35.0)	NR	59.0 ± 19.5 (18.7–94.6)	0	0	1
Guicherd et al. [31]	2017	France	Prospective case series	Endoscopic	64	24 (37)	56.3 (33.0–78.0)	26.0 (18.4–37.7)	44.8 (35.0–60.0)	NR	17.6 (14.7–20.5)	NR	NR	0	0	2
Moreta et al. [32]	2021	Spain	Retrospective case series	Endoscopic	12	6 (50)	59.1 (40.0–72.0)	27.2 (21.3–31.5)	45.3 (33.0–64.0)	7.3 (3.0–12.0)	15.9 (5.0–23.0)	58.8 (37.0–76.0)	NR	0	0	1
Nikou et al. [33]	2023	Sweden	Retrospective case series	Endoscopic	12	4 (33)	64.4 ± 15.1	26.6 ± 4.3	44.6 (31.0–51.0)	8.7 ±4.5 (1.0–14.0)	18.7 ±6.8 (11.0–37.0)	NR	NR	0	0	2
O'Sullivan et al. [34]	2007	Australia	Retrospective case series	Open tenotomy	15	4 (27)	55.5 (33.0–75.0)	NR		NR	NR	58.0 (44.0–70.0)	NR	NR	NR	NR
Schoof et al. [35]	2017	Germany	Retrospective case series	Revision	12	7 (58)	63.5 (44.0–71.0)	NR	50.1 (38.0–58.0)	NR	NR	56.0 (46.0–72.0)	NR	NR	NR	NR
Valenzuela et al. [36]	2021	Chile	Retrospective case series	Endoscopic	35	15 (43)	62.0 ± 10.3 (40.0–84.0)	28.7 ± 4.9 (20.3–41.1)		NR	NR	NR	NR	0	0	0
Viamont‐Guerra et al. [37]	2021	France	Retrospective case series	Endoscopic	50	16 (50)	60.8 ± 10.5 (35.5–80.3)	26.2 ± 4.8 (18.2–39.2)	46.1 ±7.0 (25.0–60.0)	6.9 ± 5.0 (0.0–20.5)	15.0 ±8.6 (0.0–31.6)	NR	57.7 ± 11.5 (31.9–81.4)	0	0	0
Zimmerer et al. [38]	2022	Germany	Retrospective case series	Endoscopic	20	10 (50)	59.0 ± 27.7 (52.0–78.0)	25.7 ± 5.5 (20.4–34.5)	NR	5.5 ± 1.8 (2.0–8.0)	NR	NR	31.2 ± 9.8 (17.6–47.3)	0	0	2
	Endoscopic tenotomy				273	107 (39)	63.2 (27.0–84.0)	27.5 (18.2–41.1)	44.6 (25.0–64.0)	7.0 (0.0–20.5)	16.4 (−1.0–38.0)	51.5 (32–81.8)	58.0 (17.6–94.6)	2	1	10
	Acetabular cup revision				95	42 (44)	64.0 (29.0–85.0)	27.5 (18.0–45.0)	50.0 (29.0–75.0)	9.0 (2.0–22.0)	8.0 (−10.0–35.0)	58.0 (46.0–85.0)	NR	NR	NR	NR
	Open tenotomy				29	7 (24)	62.0 (33.0–81.0)	NR	NR	8.0 (3.0–18.0)	NR	56.0 (44.0–70.0)	NR	NR	NR	NR
	Conservative management				28	10 (36)	69.0 (37.0–78.0)	NR	NR	8.0 (2.0–18.0)	NR	57.0 (50.0–68.0)	NR	N/A	N/A	N/A
	Total				425	166 (39)	63.9 (27.0–85.0)	27.5 (18.0–45.0)	45.7 (25.0–75.0)	7.5 (0.0–22.0)	14.3 (−10.0–38.0)	55.7 (32.0–85.0)	58.0 (17.6–94.6)	2	1	10

Abbreviations: BMI, body mass index; HHS, Harris hip score; mHHS, modified Harris Hip Score; N/A, not applicable; NR, not reported; SD, standard deviation.

### 3.3 Quality Assessment

Of the 15 included studies, 10 were assessed as having a low risk of bias [24, 25, 26, 27, 30, 31, 32, 34, 37, 38] and five had a moderate risk of bias [28, 29, 33, 35, 36] (Table 3). The level of evidence for each outcome parameter is detailed in Table 4.

**TABLE 3:** Risk of bias assessment of non‐randomized studies of interventions.

Author	Pre‐intervention		At intervention	Post‐intervention				Overall risk of bias
	Bias due to confounding	Bias in selection of participants into the study	Bias in classification of interventions	Bias due to deviations from intended interventions	Bias due to missing data	Bias in measurement of outcomes	Bias in selection of the reported result	
Batailler et al. [24]	Low	Moderate	Low	Low	Low	Low	Medium	Low
Bonano et al. [25]	Low	Moder	Low	Low	Low	Low	Medium	Low
Chalmers et al. [26]	Medium	Medium	Low	Low	Low	Low	Medium	Low
Di Benedetto et al. [27]	Medium	Low	Low	Low	Low	Low	Low	Low
Dora et al. [28]	Medium	Medium	Low	Low	Low	Low	Medium	Medium
Filanti et al. [29]	Medium	Medium	Low	Low	Low	Low	Medium	Medium
Finsterwald et al. [30]	Low	Low	Low	Low	Low	Low	Medium	Low
Guicherd et al. [31]	Low	Low	Low	Low	Low	Low	Low	Low
Moreta et al. [32]	Low	Medium	Low	Low	Low	Low	Medium	Low
Nikou et al. [33]	Medium	Medium	Low	Low	Low	Low	Medium	Medium
O'Sullivan et al. [34]	Medium	Low	Low	Low	Low	Low	Medium	Low
Schoof et al. [35]	Medium	Medium	Low	Low	Low	Low	Medium	Medium
Valenzuela et al. [36]	Medium	Medium	Medium	Low	Medium	Low	Medium	Medium
Viamont‐Guerra et al. [37]	Low	Low	Low	Low	Low	Low	Medium	Low
Zimmerer et al. [38]	Low	Medium	Low	Low	Low	Low	Medium	Low

*Note*: (green): Low risk of bias; (yellow): Moderate risk of bias; (red): High risk of bias.

**TABLE 4:** Level of evidence assessment according to GRADE recommendations.

No. of studies	Risk of bias	Inconsistency	Indirectness	Imprecision	Other considerations	Quality of evidence
**HHS post‐intervention**						
7	Moderate	Serious inconsistency	No serious indirectness	No serious imprecision	—	Moderate
**HHS difference**						
7	Moderate	Serious inconsistency	No serious indirectness	No serious imprecision	—	Moderate
**Functional MCID post‐intervention**						
15	Moderate	Serious inconsistency	No serious indirectness	No serious imprecision	—	Moderate

Abbreviations: GRADE, Grading of Recommendations, Assessment, Development, and Evaluation; HHS, Harris Hip Score; MCID, Minimal clinically important difference.

### 3.4 Multilevel Meta‐Analysis

#### 3.4.1 HHS Post‐Intervention

**FIGURE 2:**
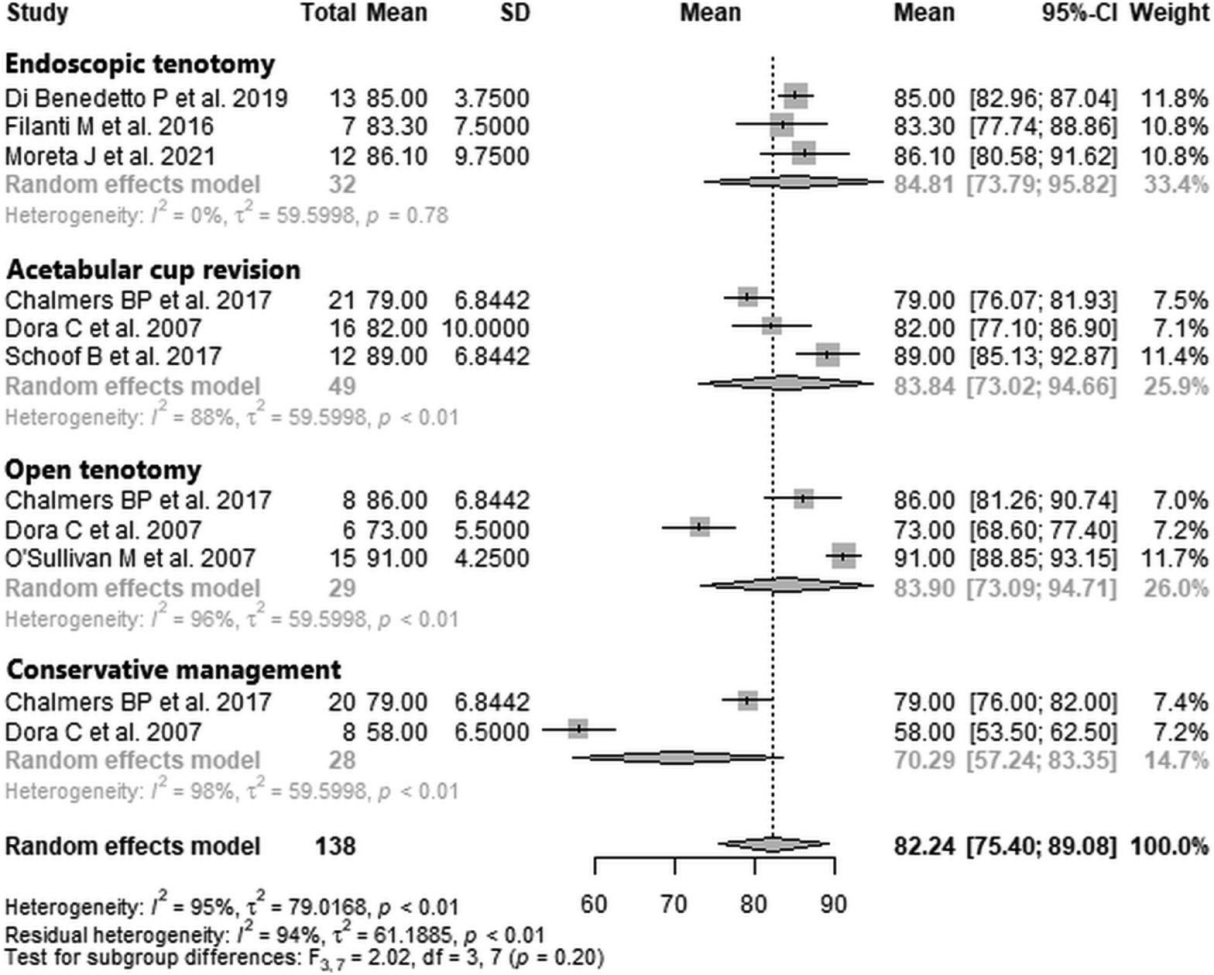
Forest plot of HHS post‐intervention. CI, confidence interval; HHS, Harris Hip Score; SD, standard deviation.

Data from 138 patients from seven studies [26, 27, 28, 29, 32, 34, 35] were pooled (Figure 2, Table 5), with the endoscopic tenotomy subgroup consisting of 32 patients from three studies [27, 29, 32], the acetabular cup revision subgroup consisting of 49 patients from three studies [26, 28, 35], the open tenotomy subgroup consisting of 29 patients from three studies [26, 28, 34], and the conservative management subgroup consisting of 28 patients from two studies [26, 28]. The mean HHS post‐intervention of the entire patient group was 82.2 points (mean: 82.2; CIs: 75.4–89.1; *I* ^2^ = 95%; *τ* ^2^ = 79.0; *p* < 0.01). The mean HHS post‐intervention of the endoscopic tenotomy subgroup was 84.8 points (mean: 84.8; CIs: 73.8–95.8; *I* ^2^ = 0%; *τ* ^2^ = 59.6; *p* = 0.78); the mean HHS post‐intervention of the acetabular cup revision subgroup was 83.8 points (mean: 83.8; CIs: 73.0–94.7; *I* ^2^ = 88%; *τ* ^2^ = 59.6; *p* < 0.01); the mean HHS post‐intervention of the open tenotomy subgroup was 83.9 points (mean: 83.9; CIs: 73.1–94.7; *I* ^2^ = 96%; *τ* ^2^ = 59.6; *p* < 0.01); the mean HHS post‐intervention of the conservative management subgroup was 70.3 points (mean: 70.3; CIs: 57.2–83.4; *I* ^2^ = 98%; *τ* ^2^ = 59.6; *p* < 0.01). The test for subgroup differences showed no statistically significant difference between the four treatment subgroups in HHS post‐intervention (*F* = 2.0; df = 3, 7; *p* = 0.20).

**TABLE 5:** Overview of the most important results of the multilevel meta‐analysis.

	Number of studies	Number of patients	Mean	Lower CI	Upper CI	*τ* ^2^	*I* ^2^	Heterogeneity *p*	Difference *p*
HHS post‐intervention									
Total	11	138	82.2	75.4	89.1	79.0	0.95	< 0.0001***	0.2003
Endoscopic tenotomy	3	32	84.8	73.8	95.8	59.6	0.00	0.7757	—
Acetabular cup revision	3	49	83.8	73.0	94.7	59.6	0.88	0.0003***	—
Open tenotomy	3	29	83.9	73.1	94.7	59.6	0.96	< 0.0001***	—
Conservative management	2	28	70.3	57.2	83.4	59.6	0.98	< 0.0001***	—
HHS difference									
Total	10	118	25.8	16.8	34.7	117.7	0.94	< 0.0001***	0.2168
Endoscopic tenotomy	3	32	28.9	14.1	41.9	84.6	0.91	< 0.0001***	—
Acetabular cup revision	3	49	25.6	12.0	39.1	84.6	0.84	0.0017**	—
Open tenotomy	3	29	26.9	13.4	40.4	84.6	0.95	< 0.0001***	—
Conservative management	1	8	3.0	−20.3	26.2	84.6	—	—	—
Functional MCID post‐intervention									
Total	19	425	9.0	7.6	10.5	7.2	1.00	< 0.0001***	0.4220
Endoscopic tenotomy	10	273	9.0	7.1	10.9	7.7	1.00	< 0.0001***	—
Acetabular cup revision	4	95	9.2	6.4	11.9	7.7	1.00	< 0.0001***	—
Open tenotomy	3	29	9.7	6.8	12.6	7.7	0.96	< 0.0001***	—
Conservative management	2	28	8.1	5.1	11.1	7.7	0.98	< 0.0001***	—

*Note*: *, significant; **, very significant; ***, highly significant.

Abbreviations: CI, confidence interval; HHS, Harris Hip Score; MCID, minimal clinically important difference.

#### 3.4.2 HHS Difference

**FIGURE 3:**
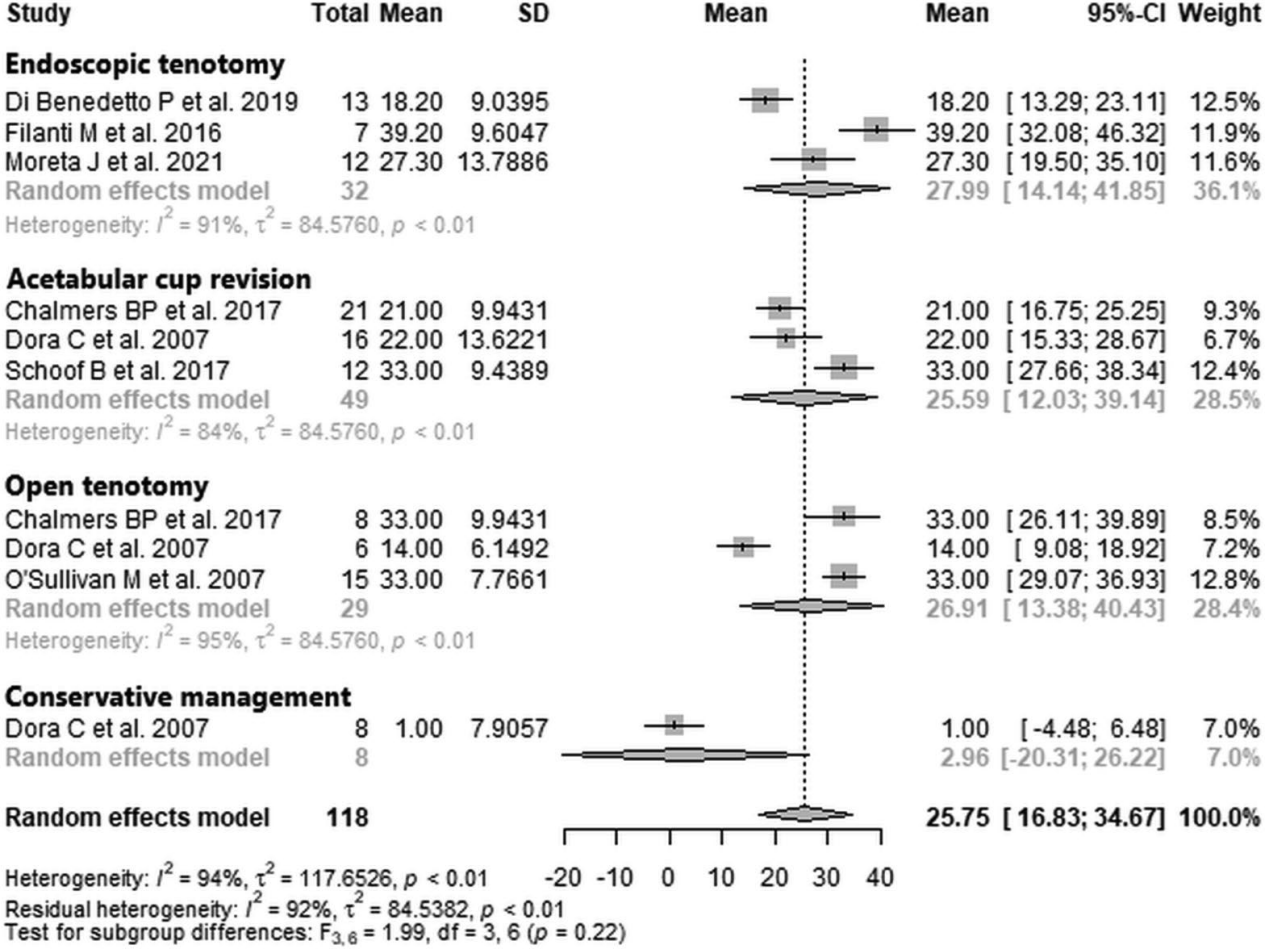
Forest plot of HHS difference. CI, confidence interval; HHS, Harris Hip Score; SD, standard deviation.

Data from 118 patients from seven studies [26, 27, 28, 29, 32, 34, 35] were pooled (Figure 3, Table 5), with the endoscopic tenotomy subgroup consisting of 32 patients from three studies [27, 29, 32], the acetabular cup revision subgroup consisting of 49 patients from three studies [26, 28, 35], the open tenotomy subgroup consisting of 29 patients from three studies [26, 28, 34], and the conservative management subgroup consisting of 8 patients from one study [28]. The mean HHS difference of the entire patient group was 25.75 points (mean: 25.75; CIs: 16.8–34.7; *I* ^2^ = 94%; *τ* ^2^ = 117.7; *p* < 0.01). The mean HHS difference of the endoscopic tenotomy subgroup was 28.0 points (mean: 28.0; CIs: 14.1–41.9; *I* ^2^ = 91%; *τ* ^2^ = 84.6; *p* < 0.01); the mean HHS difference of the acetabular cup revision subgroup was 25.6 points (mean: 25.6; CIs: 12.0–39.1; *I* ^2^ = 84%; *τ* ^2^ = 84.6; *p* < 0.01); the mean HHS difference of the open tenotomy subgroup was 26.9 points (mean: 26.9; CIs: 13.4–40.4; *I* ^2^ = 95%; *τ* ^2^ = 84.6; *p* < 0.01); the mean HHS difference of the conservative management subgroup was 3.0 points (mean: 3.0; CIs: −20.3 to 26.2). The test for subgroup differences showed no statistically significant difference between the four treatment subgroups in HHS difference (*F* = 2.0; df = 3, 6; *p* = 0.22).

#### 3.4.3 Functional MCID Post‐Intervention

**FIGURE 4:**
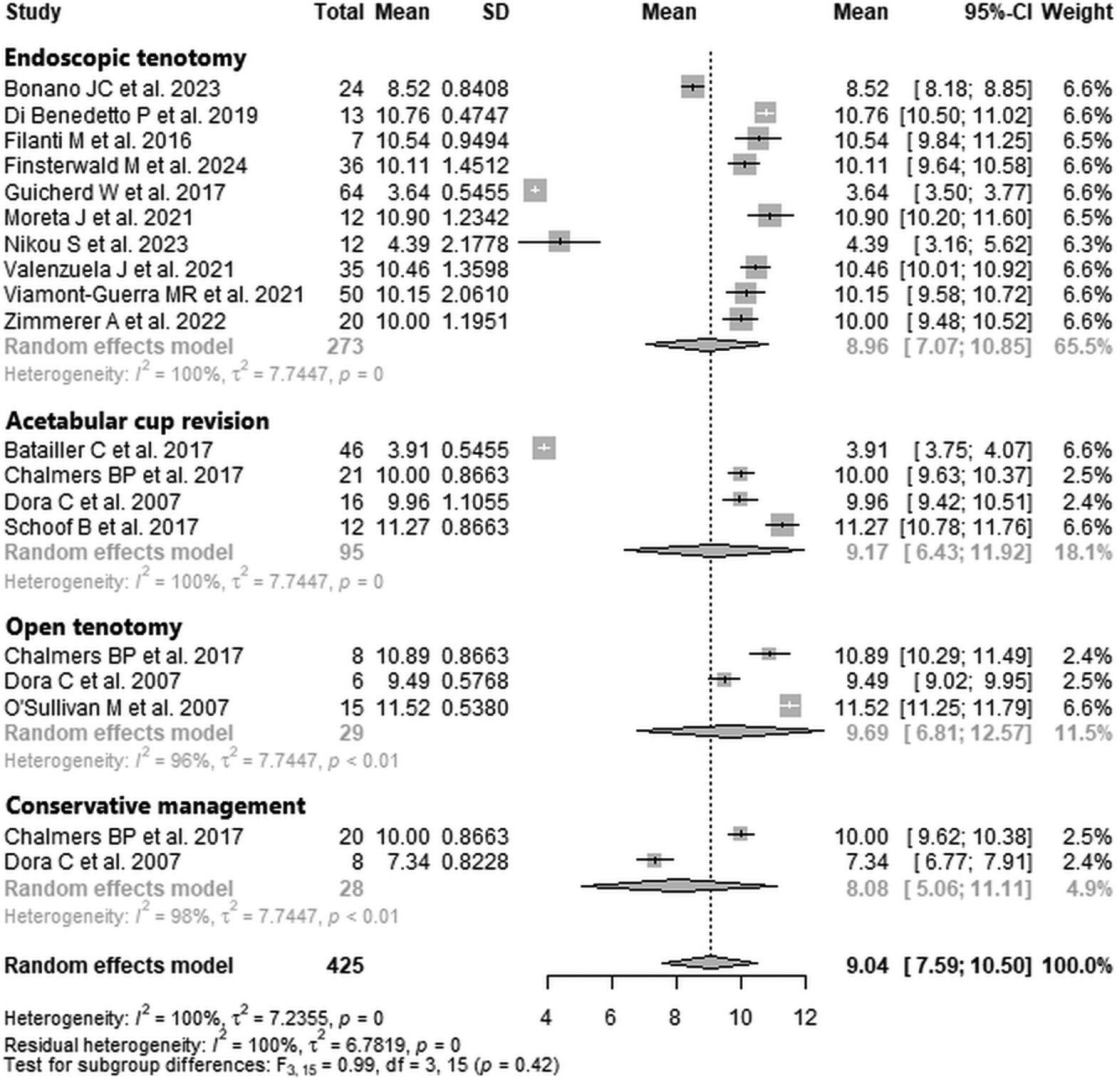
Forest plot of functional MCID post‐intervention. CI, confidence interval; MCID, minimal clinically important difference; SD, standard deviation.

Data from 425 patients from 15 studies [24, 25, 26, 27, 28, 29, 30, 31, 32, 33, 34, 35, 36, 37, 38] were pooled (Figure 4, Table 5), with the endoscopic tenotomy subgroup consisting of 273 patients from 10 studies [25, 27, 29, 30, 31, 32, 33, 36, 37, 38], the acetabular cup revision subgroup consisting of 95 patients from four studies [24, 26, 28, 35], the open tenotomy subgroup consisting of 29 patients from three studies [26, 28, 34], and the conservative management subgroup consisting of 28 patients from two studies [26, 28]. The functional MCID post‐intervention of the entire patient group was 9.0 points (mean: 9.0; CIs: 7.6–10.5; *I* ^2^ = 100%; *τ* ^2^ = 7.2; *p* = 0.00). The mean functional MCID post‐intervention of the endoscopic tenotomy subgroup was 9.0 points (mean: 9.0; CIs: 7.1–10.9; *I* ^2^ = 100%; *τ* ^2^ = 7.7; *p* = 0.00); the mean functional MCID post‐intervention of the acetabular cup revision subgroup was 9.2 points (mean: 9.2; CIs: 6.4–11.9; *I* ^2^ = 100%; *τ* ^2^ = 7.7; *p* = 0.00); the mean functional MCID post‐intervention of the open tenotomy subgroup was 9.7 points (mean: 9.7; CIs: 6.8–12.6; *I* ^2^ = 96%; *τ* ^2^ = 7.7; *p* < 0.01); the mean functional MCID post‐intervention of the conservative management subgroup was 8.1 points (mean: 8.1; CIs: 5.1–11.1; *I* ^2^ = 98%; *τ* ^2^ = 7.7; *p* < 0.01). The test for subgroup differences showed no statistically significant difference between the four treatment subgroups in functional MCID post‐intervention (*F* = 1.0; df = 3, 2; *p* = 0.42).

Additional forest plots for other functional and pain outcome parameters, and recurrence, revision, and complication rates are available in the Supporting Information (Figures [Link], [Link]). Details of the meta‐regression analysis of the examined outcome parameters (HHS post‐intervention, HHS difference, functional MCID post‐intervention) in relation to patient demographic independent variables (sex, age, BMI) are shown in Table 6. The results show no statistically significant differences, as presented in the last column of the table.

**TABLE 6:** Meta‐regression analysis of independent demographic and study‐related variables.

Outcome	Predictor	Primary studies	*β*	Lower CI	Upper CI	*p*
HHS post‐intervention	Male sex (in %)	11	−0.04	−0.33	0.26	0.78
	Age	11	−0.05	−1.26	1.15	0.92
	BMI	1				
HHS difference	Male sex (in %)	10	−0.12	−0.45	0.22	0.45
	Age	10	−0.35	−1.98	1.28	0.64
	BMI	1				
Functional MCID post‐intervention	Male sex (in %)	19	0.01	−0.04	0.05	0.79
	Age	19	0.04	−0.13	0.20	0.63
	BMI	9	0.50	−1.19	2.19	0.51

Abbreviations: BMI, body mass index; CI, confidence interval; HHS, Harris hip score; MCID, minimal clinically important difference.

## 4 Discussion

This systematic review and multilevel meta‐analysis, including 15 studies with 425 patients, found that all four treatment options for IPI after THA resulted in functional improvements, with no statistically significant differences between surgical and conservative interventions. However, conservative management exhibited the lowest mean HHS post‐intervention (70.3 points), suggesting it may be less effective than surgical options. Surgical treatments, including endoscopic tenotomy, acetabular cup revision, and open tenotomy, showed comparable mean HHS scores above 80, indicating good functional outcomes. Despite these findings, substantial heterogeneity was observed, particularly in the conservative management and open tenotomy groups, highlighting the need for further high‐quality research with standardized methodologies.

### 4.1 HHS Post‐Intervention Outcomes

Across all treatments, patients demonstrated a mean post‐intervention HHS of 82.2 points, with no statistically significant differences detected among the treatment subgroups. Although HHS values above 80 generally indicate good hip function, it is essential to recognize the substantial heterogeneity in these results, as evidenced by the high *I* ^2^ values for most subgroups, particularly in the conservative management group (*I* ^2^ = 98%). The heterogeneity could be attributed to differences in patient demographics, surgical techniques, and follow‐up durations across studies. Notably, the conservative management subgroup exhibited the lowest mean HHS (70.3 points), suggesting that this noninvasive approach may result in relatively poorer outcomes compared to surgical options.

The lack of significant inter‐group differences in HHS scores across treatments might suggest comparable efficacy among the surgical options, particularly endoscopic tenotomy, acetabular cup revision, and open tenotomy. This finding may be significant in contexts where one surgical approach is preferable due to patient preference, surgeon expertise, or resource availability. However, the relatively lower outcome in the conservative management group underscores the potential benefit of surgical intervention, as patients in this group may experience less favorable functional recovery.

### 4.2 HHS Difference Post‐Intervention Outcomes

The HHS difference, which represents the subtracted value between post‐ and pre‐intervention HHS, further supports the efficacy of the surgical options over conservative management. The endoscopic tenotomy, acetabular cup revision, and open tenotomy subgroups all demonstrated substantial improvements (mean HHS differences of 28.0, 25.6, and 26.9 points, respectively). In contrast, the conservative management group had a negligible mean HHS difference of 3.0, with confidence intervals that include negative values, indicating minimal or no improvement for these patients. This contrast highlights the benefit of surgical intervention, as patients undergoing any of the surgical options achieved clinically meaningful functional gains.

The subgroup analysis reveals no significant statistical difference among the surgical interventions in terms of HHS improvement, which might suggest that the type of surgery performed may be less critical than the fact of receiving a surgical intervention itself. However, a future multilevel meta‐analysis of a larger cohort of patients may be expected to show significant differences.

### 4.3 Functional MCID Post‐Intervention Outcomes

Conversion of the various functional scores to their MCIDs allowed us to combine HHS, mHHS, iHOT, OHS, and SANE into a single functional MCID outcome parameter, which gave more consistent results with a total of 15 included studies [24, 25, 26, 27, 28, 29, 30, 31, 32, 33, 34, 35, 36, 37, 38] with 425 patients. The functional MCID, which reflects the minimal score improvement perceived as beneficial by patients, ranged from 8.1 in the conservative management group to 9.7 in the revision and open tenotomy groups. Again, the mean of the conservative management group was lower compared to the surgical groups, suggesting that conservative management may be less effective in achieving optimal patient‐perceived improvements.

While conservative management showed the lowest mean functional MCID and HHS scores compared to surgical options, our ability to explore its potential role in specific patient subgroups is limited by the small sample size in this group. Future studies with larger, more balanced cohorts may help elucidate whether certain subgroups of patients could benefit more from conservative approaches.

Interestingly, despite the heterogeneity observed in HHS outcomes, the functional MCID scores were relatively similar across surgical subgroups. This minimal variation in mean values suggests that, while surgical outcomes can be variable, patients generally perceive a similar level of functional improvement following surgery.

### 4.4 Strengths

This systematic review and multilevel meta‐analysis provides a comprehensive evaluation of treatment options for IPI after THA, including endoscopic tenotomy, acetabular cup revision, open tenotomy, and conservative management. By analyzing functional outcomes using standardized measures (HHS, mHHS, iHOT, OHS, SANE) and considering MCIDs, this study offers valuable insights for clinical decision‐making and patient expectations.

To improve future research, larger, well‐designed studies with homogeneous patient populations and standardized outcome reporting are needed. Incorporating patient‐reported outcomes, such as quality of life and satisfaction, would provide a more holistic assessment of treatment effectiveness. Additionally, standardized long‐term follow‐up data would enhance the evaluation of treatment safety and durability over time.

### 4.5 Limitations

A key limitation is the small sample size in the open tenotomy and conservative management subgroups, which affects statistical power and contributes to significant heterogeneity (high *I* ^2^ values). While a random‐effects model was used to account for variability, the high heterogeneity suggests differences in patient demographics, surgical techniques, and follow‐up durations across studies. Although a meta‐regression analysis was conducted, it did not identify statistically significant factors influencing this heterogeneity, indicating that variability may stem from factors not captured in the primary studies.

Additionally, inconsistencies in reporting surgical techniques and surgeon expertise limit the ability to assess their influence on outcomes. Variability in postoperative care, rehabilitation protocols, and institutional differences may also contribute to differing results. Future studies should aim to standardize these variables to improve comparability and reliability.

The conservative management group exhibited particularly high heterogeneity, reflecting a lack of standardized treatment protocols across studies. Further research should refine non‐surgical approaches and identify patient subgroups most likely to benefit from conservative care. Given these limitations, the findings should be interpreted with caution, and more homogeneous studies are needed to strengthen conclusions.

### 4.6 Clinical Implications and Future Research

Surgical interventions, including endoscopic tenotomy, acetabular cup revision, and open tenotomy, generally yield favorable functional outcomes, supporting their role as effective treatment options. Since functional outcomes appear comparable across surgical methods, factors such as surgeon expertise, patient preference, and clinical context should guide treatment choices. In contrast, conservative management may offer limited benefits and should be considered primarily when surgery is contraindicated or declined.

Clinical decision‐making should factor in patient‐specific variables such as age, activity level, and comorbidities. Younger, active patients may benefit more from surgical approaches, while older or higher‐risk individuals may be better suited to conservative strategies. Further research should refine conservative treatment protocols to improve their effectiveness and identify patient subgroups most likely to benefit from non‐surgical approaches.

## 5 Conclusion

In conclusion, this multilevel meta‐analysis indicates that surgical interventions, including endoscopic tenotomy, acetabular cup revision, and open tenotomy, are effective in achieving meaningful functional improvements in IPI patients. Despite some variability in outcomes, patients undergoing surgery generally reported improvements that align with functional MCID thresholds. While conservative management was the least effective of all treatment groups, the differences did not reach statistical significance.

## Author Contributions

N.R. and M.V. performed the literature search. R.H. and N.R. performed the statistics. N.R. created tables and figures. N.R. wrote the manuscript. All authors supervised the whole process and read the final version.

## Ethics Statement

The authors have nothing to report.

## Conflicts of Interest

The authors declare no conflicts of interest.

## Supporting information


**FIGURE S1.** Forest plot HHS preoperatively.

**FIGURE S2.** Forest plot HHS postoperatively.

**FIGURE S3.** Forest plot mHHS preoperatively.

**FIGURE S4.** Forest plot mHHS postoperatively.

**FIGURE S5.** Forest plot mHHS MCID.

**FIGURE S6.** Forest plot mHHS difference.

**FIGURE S7.** Forest plot OHS preoperatively.

**FIGURE S8.** Forest plot OHS postoperatively.

**FIGURE S9.** Forest plot OHS MCID.

**FIGURE S10.** Forest plot OHS difference.

**FIGURE S11.** Forest plot iHOT 12 postoperatively.

**FIGURE S12.** Forest plot iHOT 12 MCID.

**FIGURE S13.** Forest plot VAS preoperatively.

**FIGURE S14.** Forest plot VAS postoperatively.

**FIGURE S15.** Forest plot VAS MCID.

**FIGURE S16.** Forest plot VAS difference.

**FIGURE S17.** Forest plot Pain MCID.

**FIGURE S18.** Forest plot Complications.

**FIGURE S19.** Forest plot Recurrence.

**FIGURE S20.** Forest plot Revision.

**TABLE S1.** PRISMA 2020 checklist.

**TABLE S2.** Data extraction sheet.

## Data Availability

Raw data extraction sheet is available in the supplement.

## References

[1] P. Söderman , H. Malchau , and P. Herberts , “Outcome After Total Hip Arthroplasty: Part I. General Health Evaluation in Relation to Definition of Failure in the Swedish National Total hip Arthoplasty Register,” Acta Orthopaedica Scandinavica 71, no. 4 (2000): 354–359, 10.1080/000164700317393330. 11028882

[2] P. Söderman , H. Malchau , P. Herberts , R. Zügner , H. Regnér , and G. Garellick , “Outcome After Total Hip Arthroplasty: Part II. Disease‐Specific Follow‐Up and the Swedish National Total hip Arthroplasty Register,” Acta Orthopaedica Scandinavica 72, no. 2 (2001): 113–119, 10.1080/000164701317323345. 11372940

[3] A. A. Marth , C. Ofner , P. O. Zingg , and R. Sutter , “Quantifying Cup Overhang After Total Hip Arthroplasty: Standardized Measurement Using Reformatted Computed Tomography and Association of Overhang Distance With Iliopsoas Impingement,” European Radiology 34, no. 7 (2024): 4300–4308, 10.1007/s00330-023-10479-5. 38147169 PMC11213778

[4] M. Hardwick‐Morris , J. Twiggs , B. Miles , et al., “Determination of Preoperative Risk Factors for Iliopsoas Tendonitis After Total Hip Arthroplasty: A Simulation Study,” Journal of Orthopaedic Research 42, no. 9 (2024): 2035–2042, 10.1002/jor.25856. 38587991

[5] G. E. Lewinnek , J. L. Lewis , R. Tarr , C. L. Compere , and J. R. Zimmerman , “Dislocations After Total Hip‐Replacement Arthroplasties,” Journal of Bone and Joint Surgery. American Volume 60, no. 2 (1978): 217–220. 641088

[6] C. Carbonell‐Rosell , D. Soza , O. Pujol , M. de Albert Delás‐Vigo , A. Antón , and V. Barro , “Iliopsoas Impingement After Total Hip Arthroplasty: Does the CT‐Scan Have any Role? Our Algorithm Proposal,” Journal of Orthopaedics 34 (2022): 137–141, 10.1016/j.jor.2022.08.023. 36072762 PMC9441293

[7] R. Torres‐Eguia , L. E. Betancourt , J. Mas Martinez , and J. Sanz‐Reig , “Severe Weakness of Hip Flexor After Iliopsoas Tenotomy: Two Case Reports,” Hip & Pelvis 32, no. 2 (2020): 112–117, 10.5371/hp.2020.32.2.112. 32566543 PMC7295613

[8] P. J. Duwelius , R. D. Southgate , J. P. Crutcher, Jr. , et al., “Registry Data Show Complication Rates and Cost in Revision Hip Arthroplasty,” Journal of Arthroplasty 38, no. 7S (2023): S29–S33, 10.1016/j.arth.2023.04.050. 37121489

[9] R. Prill , A. Królikowska , M. Enes Kayaalp , N. Ramadanov , J. Karlsson , and M. T. Hirschmann , “Enhancing Research Methods: The Role of Systematic and Scoping Reviews in Orthopaedics, Sports Medicine and Rehabilitation,” Journal of Experimental Orthopaedics 11, no. 4 (2024): e70069, 10.1002/jeo2.70069. 39502322 PMC11534858

[10] R. Coulomb , B. Nougarede , E. Maury , P. Marchand , O. Mares , and P. Kouyoumdjian , “Arthroscopic Iliopsoas Tenotomies: A Systematic Review of Surgical Technique and Outcomes,” Hip International 32, no. 1 (2022): 4–11, 10.1177/1120700020970519. 33226846

[11] R. Giai Via , M. Giachino , A. Elzeiny , et al., “Arthroscopic and Endoscopic Techniques for Iliopsoas Release in THA Are Safe and Effective: A Systematic Review of the Literature,” European Journal of Orthopaedic Surgery and Traumatology 34, no. 6 (2024): 2845–2857, 10.1007/s00590-024-04042-1. 38977435 PMC11377508

[12] R. S. O'Connell , D. S. Constantinescu , D. J. Liechti , J. J. Mitchell , and A. R. Vap , “A Systematic Review of Arthroscopic Versus Open Tenotomy of Iliopsoas Tendonitis After Total Hip Replacement,” Arthroscopy 34, no. 4 (2018): 1332–1339, 10.1016/j.arthro.2017.10.051. 29361421

[13] J. Shapira , S. L. Chen , N. M. Wojnowski , et al., “Outcomes of Nonoperative Management, Iliopsoas Tenotomy, and Revision Arthroplasty for Iliopsoas Impingement After Total Hip Arthroplasty: A Systematic Review,” Journal of Arthroplasty 34, no. 9 (2019): 2184–2191, 10.1016/j.arth.2019.04.067. 31147246

[14] M. J. Page , J. E. McKenzie , P. M. Bossuyt , et al., “The PRISMA 2020 Statement: An Updated Guideline for Reporting Systematic Reviews,” BMJ 372 (2021): n71, 10.1136/bmj.n71. 33782057 PMC8005924

[15] N. Ramadanov , M. Voss , R. Hable , et al., “Postoperative Harris Hip Score Versus Harris Hip Score Difference in Hip Replacement: What to Report?,” Orthopaedic Surgery 17, no. 1 (2025): 3–21, 10.1111/os.14272. 39434235 PMC11735366

[16] M. Ostojic , P. W. Winkler , J. Karlsson , R. Becker , and R. Prill , “Minimal Clinically Important Difference: Don't Just Look at the *p*‐Value,” Knee Surgery, Sports Traumatology, Arthroscopy 31, no. 10 (2023): 4077–4079, 10.1007/s00167-023-07512-x. 37460662

[17] B. U. Nwachukwu , B. Chang , B. Z. Rotter , B. T. Kelly , A. S. Ranawat , and D. H. Nawabi , “Minimal Clinically Important Difference and Substantial Clinical Benefit After Revision Hip Arthroscopy,” Arthroscopy 34, no. 6 (2018): 1862–1868, 10.1016/j.arthro.2018.01.050. 29653791

[18] B. U. Nwachukwu , K. Fields , B. Chang , D. H. Nawabi , B. T. Kelly , and A. S. Ranawat , “Preoperative Outcome Scores Are Predictive of Achieving the Minimal Clinically Important Difference After Arthroscopic Treatment of Femoroacetabular Impingement,” American Journal of Sports Medicine 45, no. 3 (2017): 612–619, 10.1177/0363546516669325. 27765733

[19] R. Thomeé , P. Jónasson , K. Thorborg , et al., “Cross‐Cultural Adaptation to Swedish and Validation of the Copenhagen Hip and Groin Outcome Score (HAGOS) for Pain, Symptoms and Physical Function in Patients With Hip and Groin Disability due to Femoro‐Acetabular Impingement,” Knee Surgery, Sports Traumatology, Arthroscopy 22, no. 4 (2014): 835–842, 10.1007/s00167-013-2721-7. 24146052

[20] D. J. Beard , K. Harris , J. Dawson , et al., “Meaningful Changes for the Oxford Hip and Knee Scores After Joint Replacement Surgery,” Journal of Clinical Epidemiology 68, no. 1 (2015): 73–79, 10.1016/j.jclinepi.2014.08.009. 25441700 PMC4270450

[21] G. Nazari , P. Bobos , S. Lu , and J. C. MacDermid , “Psychometric Properties of the Single Assessment Numeric Evaluation in Patients With Lower Extremity Pathologies. A Systematic Review,” Disability and Rehabilitation 43, no. 15 (2021): 2092–2099, 10.1080/09638288.2019.1693641. 31775536

[22] J. A. Sterne , M. A. Hernán , B. C. Reeves , et al., “ROBINS‐I: A Tool for Assessing Risk of Bias in Non‐Randomised Studies of Interventions,” BMJ 355 (2016): i4919, 10.1136/bmj.i4919. 27733354 PMC5062054

[23] G. H. Guyatt , A. D. Oxman , G. E. Vist , et al., “GRADE: An Emerging Consensus on Rating Quality of Evidence and Strength of Recommendations,” BMJ 336, no. 7650 (2008): 924–926, 10.1136/bmj.39489.470347.AD. 18436948 PMC2335261

[24] C. Batailler , N. Bonin , M. Wettstein , et al., “Outcomes of Cup Revision for Ilio‐Psoas Impingement After Total Hip Arthroplasty: Retrospective Study of 46 Patients,” Orthopaedics & Traumatology, Surgery & Research 103, no. 8 (2017): 1147–1153, 10.1016/j.otsr.2017.07.021. 28951281

[25] J. C. Bonano , K. Pierre , C. Jamero , N. A. Segovia , J. I. Huddleston , and M. R. Safran , “Endoscopic Iliopsoas Lengthening for Treatment of Recalcitrant Iliopsoas Tendinitis After Total Hip Arthroplasty,” Journal of Hip Preservation Surgery 10, no. 2 (2023): 63–68, 10.1093/jhps/hnac052. 37900893 PMC10604048

[26] B. P. Chalmers , P. K. Sculco , R. J. Sierra , R. T. Trousdale , and D. J. Berry , “Iliopsoas Impingement After Primary Total Hip Arthroplasty: Operative and Nonoperative Treatment Outcomes,” Journal of Bone and Joint Surgery. American Volume 99, no. 7 (2017): 557–564, 10.2106/JBJS.16.00244. 28375888

[27] P. Di Benedetto , G. Niccoli , S. Magnanelli , et al., “Arthroscopic Treatment of Iliopsoas Impingement Syndrome After Hip Arthroplasty,” Acta Biomed 90, no. S1 (2019): 104–109, 10.23750/abm.v90i1-S.8076. PMC6503398 30715007

[28] C. Dora , M. Houweling , P. Koch , and R. J. Sierra , “Iliopsoas Impingement After Total Hip Replacement: The Results of Non‐Operative Management, Tenotomy or Acetabular Revision,” Journal of Bone and Joint Surgery. British Volume (London) 89, no. 8 (2007): 1031–1035, 10.1302/0301-620X.89B8.19208. 17785740

[29] M. Filanti , C. Carubbi , N. Del Piccolo , N. Rani , A. Mazzotta , and D. Dallari , “The Role of Arthroscopy in the Treatment of Groin Pain After Total Hip Arthroplasty: Our Experience,” Hip International 26, no. 1 (2016): 28–33, 10.5301/hipint.5000405. 27174071

[30] M. Finsterwald , F. Mancino , G. Waters , et al., “Endoscopic Tendon Release for Iliopsoas Impingement After Total Hip Arthroplasty‐Excellent Clinical Outcomes and Low Failure Rates at Short‐Term Follow‐Up,” Arthroscopy 40, no. 3 (2024): 790–798, 10.1016/j.arthro.2023.07.040. 37544336

[31] W. Guicherd , N. Bonin , T. Gicquel , et al., “Endoscopic or Arthroscopic Iliopsoas Tenotomy for Iliopsoas Impingement Following Total Hip Replacement. A Prospective Multicenter 64‐Case Series,” Orthopaedics & Traumatology, Surgery & Research 103, no. 8S (2017): S207–S214, 10.1016/j.otsr.2017.09.007. 28917519

[32] J. Moreta , A. Cuéllar , U. Aguirre , Ó. L. Casado‐Verdugo , A. Sánchez , and R. Cuéllar , “Outside‐In Arthroscopic Psoas Release for Anterior Iliopsoas Impingement After Primary Total Hip Arthroplasty,” Hip International 31, no. 5 (2021): 649–655, 10.1177/1120700020909159. 32093495

[33] S. Nikou , I. Lindman , A. Sigurdsson , et al., “Arthroscopic Iliopsoas Tenotomy After Total Hip Arthroplasty: Safe Method for the Right Patient,” Journal of Experimental Orthopaedics 10, no. 1 (2023): 3, 10.1186/s40634-023-00568-1. 36652032 PMC9849514

[34] M. O'Sullivan , C. C. Tai , S. Richards , A. D. Skyrme , W. L. Walter , and W. K. Walter , “Iliopsoas Tendonitis a Complication After Total Hip Arthroplasty,” Journal of Arthroplasty 22, no. 2 (2007): 166–170, 10.1016/j.arth.2006.05.034. 17275628

[35] B. Schoof , O. Jakobs , S. Schmidl , et al., “Anterior Iliopsoas Impingement due to a Malpositioned Acetabular Component: Effective Relief by Surgical Cup Reorientation,” Hip International 27, no. 2 (2017): 128–133, 10.5301/hipint.5000443. 27886357

[36] J. Valenzuela and J. M. O'Donnell , “Endoscopic Treatment of Iliopsoas Impingement After Total Hip Arthroplasty: A Minimum 2‐Year Follow‐Up and Comparison of Tenotomy Performed at the Acetabular Rim Versus Lesser Trochanter,” Journal of Hip Preservation Surgery 8, no. 1 (2021): 83–89, 10.1093/jhps/hnab035. 34567604 PMC8460172

[37] M. R. Viamont‐Guerra , S. Ramos‐Pascual , M. Saffarini , and N. Bonin , “Endoscopic Tenotomy for Iliopsoas Tendinopathy Following Total Hip Arthroplasty Can Relieve Pain Regardless of Acetabular Cup Overhang or Anteversion,” Arthroscopy 37, no. 9 (2021): 2820–2829, 10.1016/j.arthro.2021.03.043. 33812032

[38] A. Zimmerer , M. Hauschild , R. Nietschke , et al., “Results After Arthroscopic Treatment of Iliopsoas Impingement After Total Hip Arthroplasty,” Archives of Orthopaedic and Trauma Surgery 142, no. 2 (2022): 189–195, 10.1007/s00402-020-03623-z. 33044706 PMC8783918

[39] O. Amellal , K. Lamraski , and M. Penasse , “Endoscopic Iliopsoas Tenotomy Following Total Hip Replacement: Retrospective Study of 19 Cases,” Journal of Arthroscopy and Joint Surgery 7, no. 3 (2020): 131–136, 10.1016/j.jajs.2020.08.003.

[40] C. D. Bell , M. B. Wagner , L. Wang , et al., “Evaluation of Endoscopic Iliopsoas Tenotomy for Treatment of Iliopsoas Impingement After Total Hip Arthroplasty,” Journal of Arthroplasty 34, no. 7 (2019): 1498–1501, 10.1016/j.arth.2019.03.030. 31005438

[41] J. E. Gédouin and D. Huten , “Technique and Results of Endoscopic Tenotomy in Iliopsoas Muscle Tendinopathy Secondary to Total Hip Replacement: A Series of 10 Cases,” Orthopaedics & Traumatology, Surgery & Research 98, no. S4 (2012): S19–S25, 10.1016/j.otsr.2012.04.009. 22591783

[42] K. Heaton and L. D. Dorr , “Surgical Release of Iliopsoas Tendon for Groin Pain After Total Hip Arthroplasty,” Journal of Arthroplasty 17, no. 6 (2002): 779–781, 10.1054/arth.2002.33570. 12216034

[43] J. Jerosch , C. Neuhäuser , and S. M. Sokkar , “Arthroscopic Treatment of Iliopsoas Impingement (IPI) After Total Hip Replacement,” Archives of Orthopaedic and Trauma Surgery 133, no. 10 (2013): 1447–1454, 10.1007/s00402-013-1806-6. 23884462

[44] M. Lahner , C. von Schulze Pellengahr , T. K. Lichtinger , et al., “The Role of Arthroscopy in Patients With Persistent Hip Pain After Total Hip Arthroplasty,” Technology and Health Care 21, no. 6 (2013): 599–606, 10.3233/THC-130761. 24284548

[45] O. Mei‐Dan , C. Pascual‐Garrido , B. Moreira , M. O. McConkey , and D. A. Young , “The Role of Hip Arthroscopy in Investigating and Managing the Painful Hip Resurfacing Arthroplasty,” Arthroscopy 32, no. 3 (2016): 459–466, 10.1016/j.arthro.2015.08.029. 26553962

[46] R. M. Nunley , J. M. Wilson , L. Gilula , J. C. Clohisy , R. L. Barrack , and W. J. Maloney , “Iliopsoas Bursa Injections Can Be Beneficial for Pain After Total Hip Arthroplasty,” Clinical Orthopaedics and Related Research 468, no. 2 (2010): 519–526, 10.1007/s11999-009-1141-y. 19851816 PMC2807015

[47] C. Pattyn , R. Verdonk , and E. Audenaert , “Hip Arthroscopy in Patients With Painful Hip Following Resurfacing Arthroplasty,” Knee Surgery, Sports Traumatology, Arthroscopy 19, no. 9 (2011): 1514–1520, 10.1007/s00167-011-1463-7. 21409469

[48] E. Tassinari , F. Castagnini , F. Mariotti , et al., “Arthroscopic Tendon Release for Iliopsoas Impingement After Primary Total Hip Arthroplasty: A Retrospective, Consecutive Series,” Hip International 31, no. 1 (2021): 125–132, 10.1177/1120700019893341. 31830823

[49] A. Van Riet , J. De Schepper , and H. P. Delport , “Arthroscopic Psoas Release for Iliopsoas Impingement After Total Hip Replacement,” Acta Orthopaedica Belgica 77, no. 1 (2011): 41–46. 21473444

[50] M. Williams and M. Ashworth , “An Operative Technique for Psoas Impingement Following Total Hip Arthroplasty: A Case Series of Day Case, Extra Articular, Arthroscopic Psoas Tenotomy,” Archives of Orthopaedic and Trauma Surgery 139, no. 2 (2019): 211–216, 10.1007/s00402-018-3029-3. 30128627

