# Supplementary figures and images for: Multilevel Meta‐Analysis of Treatment Options for Patients With Iliopsoas Impingement Syndrome After Total Hip Arthroplasty
Source: Orthop Surg. 2025 May 23;17(7):1899–912. doi: 10.1111/os.70021 (PMC12214397; doi:10.1111/os.70021)

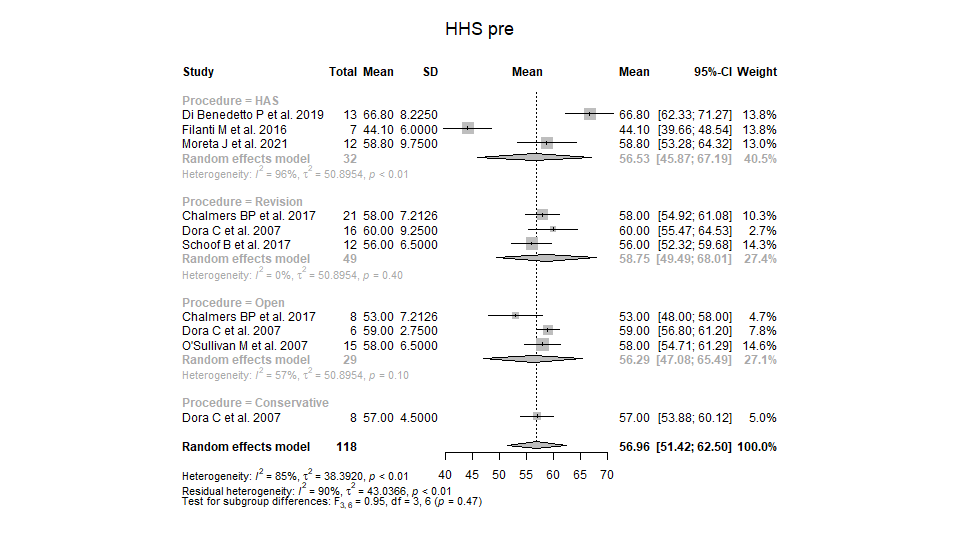

Supplement: Supplementary file 1 — FIGURE S1. Forest plot HHS preoperatively. [file OS-17-1899-s019.png]

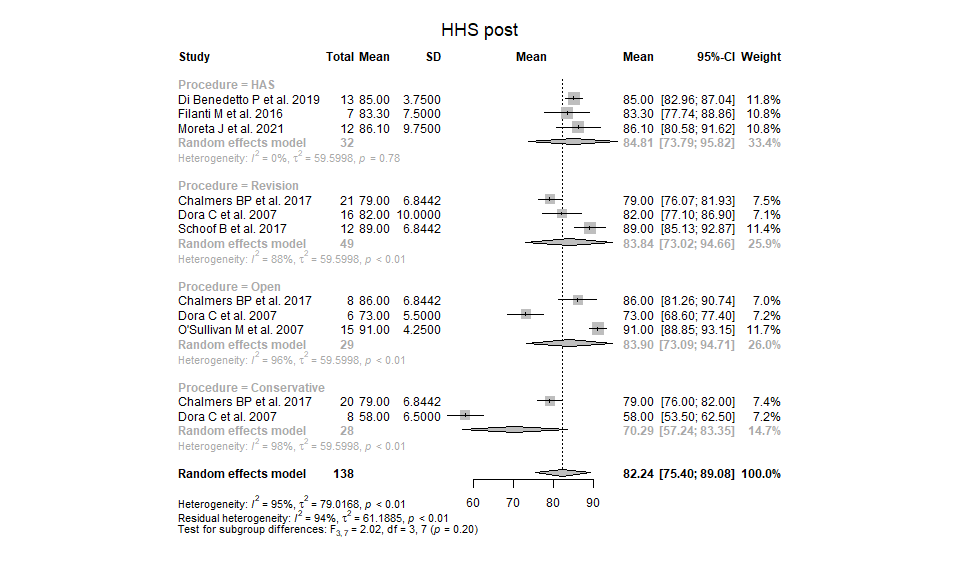

Supplement: Supplementary file 2 — FIGURE S2. Forest plot HHS postoperatively. [file OS-17-1899-s022.png]

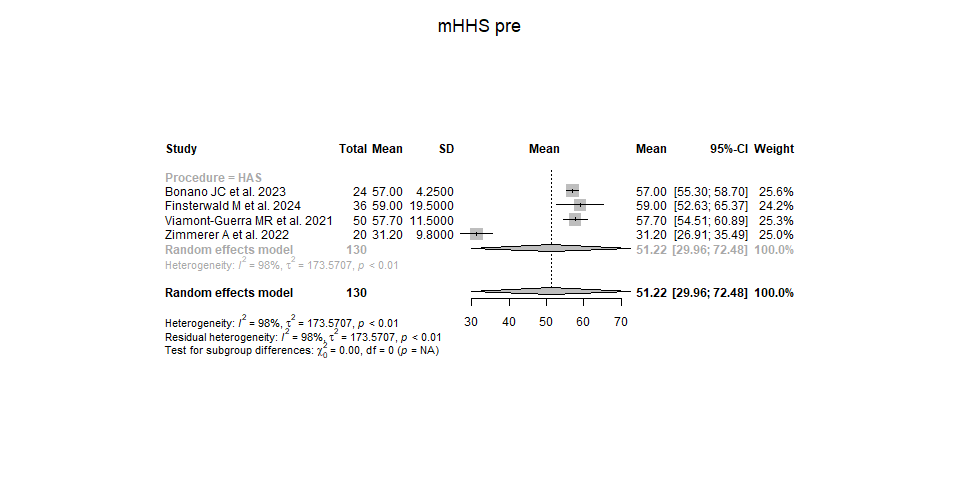

Supplement: Supplementary file 3 — FIGURE S3. Forest plot mHHS preoperatively. [file OS-17-1899-s006.png]

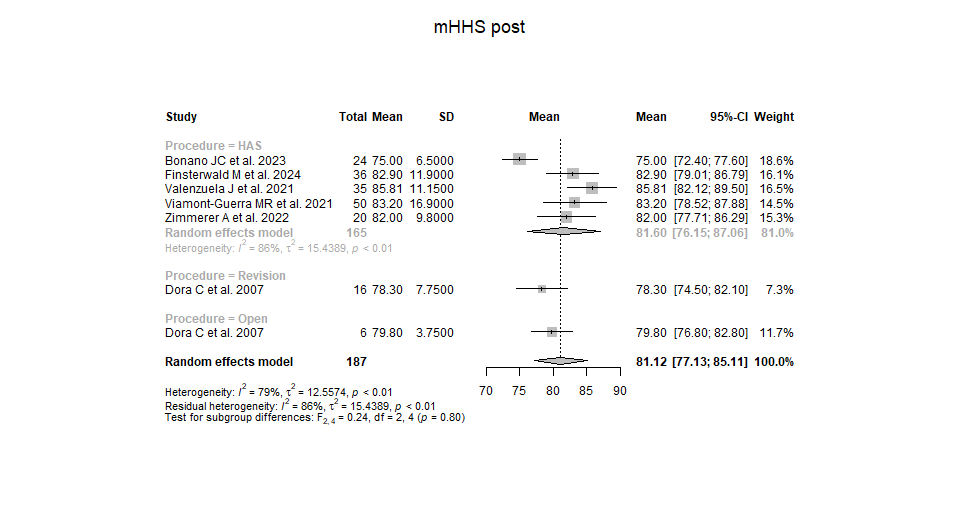

Supplement: Supplementary file 4 — FIGURE S4. Forest plot mHHS postoperatively. [file OS-17-1899-s010.png]

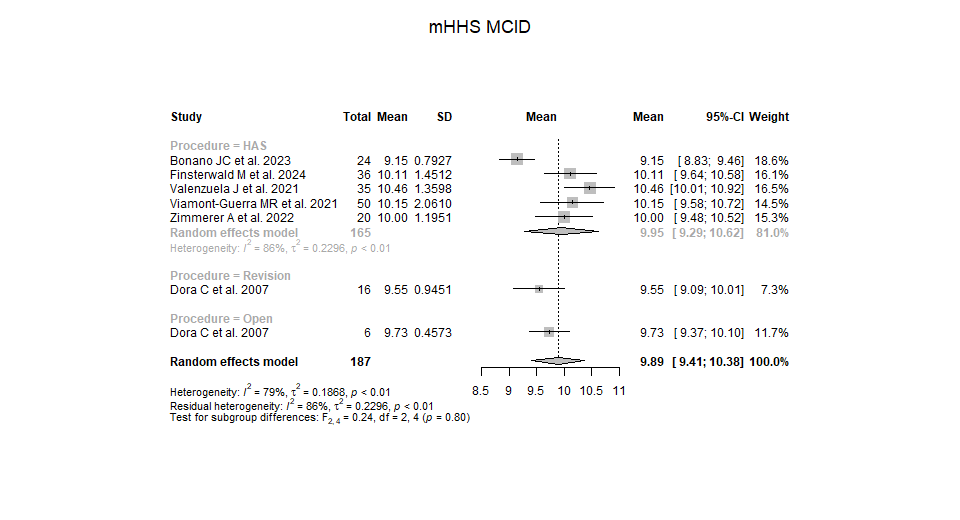

Supplement: Supplementary file 5 — FIGURE S5. Forest plot mHHS MCID. [file OS-17-1899-s002.png]

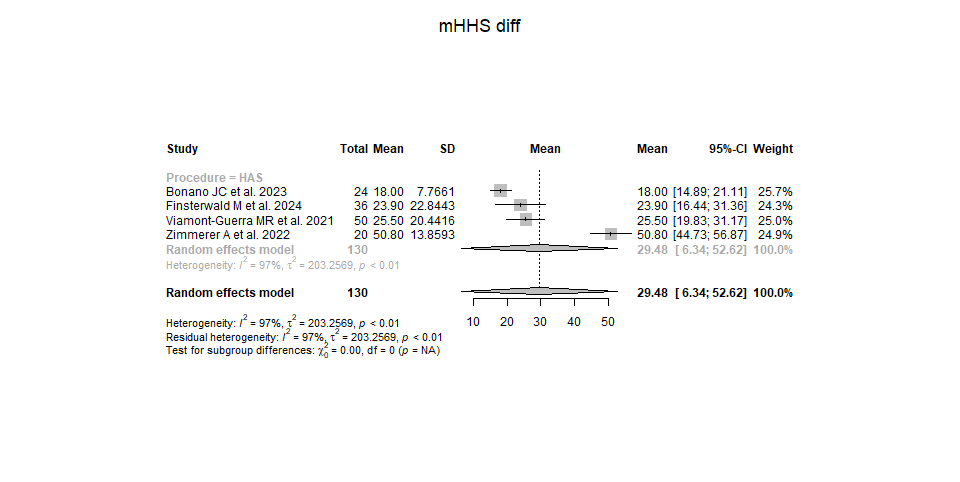

Supplement: Supplementary file 6 — FIGURE S6. Forest plot mHHS difference. [file OS-17-1899-s001.png]

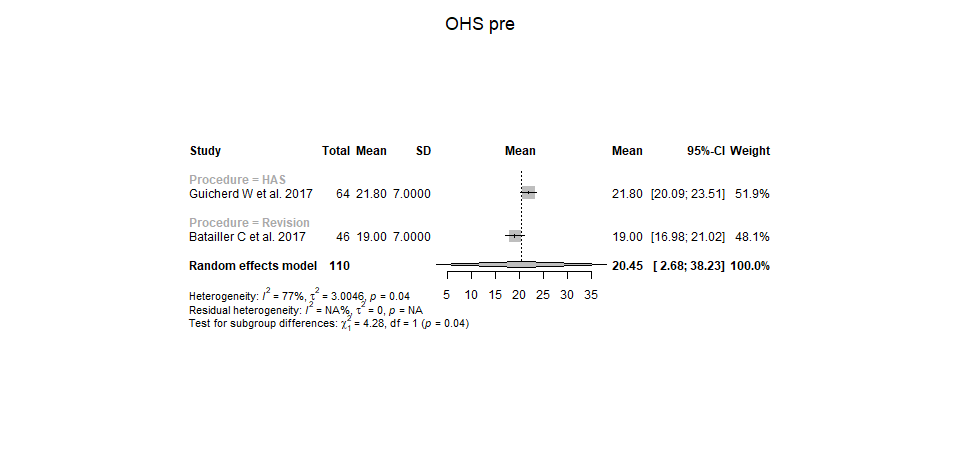

Supplement: Supplementary file 7 — FIGURE S7. Forest plot OHS preoperatively. [file OS-17-1899-s015.png]

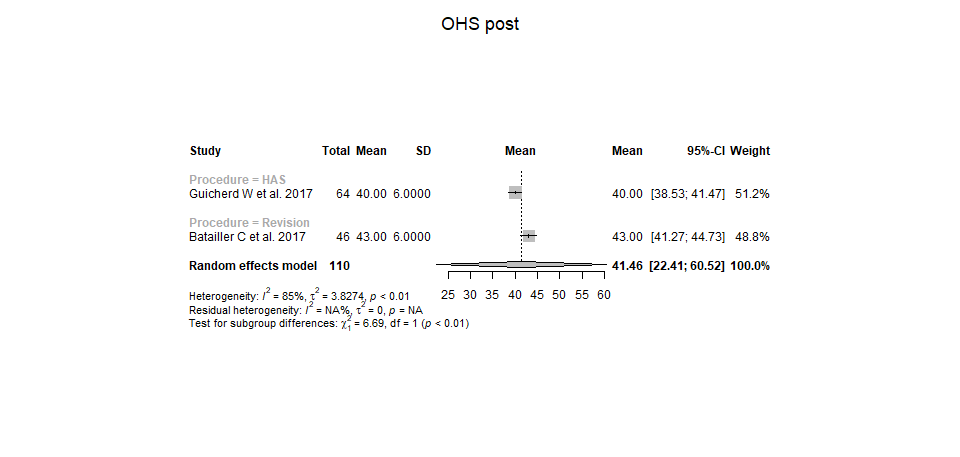

Supplement: Supplementary file 8 — FIGURE S8. Forest plot OHS postoperatively. [file OS-17-1899-s009.png]

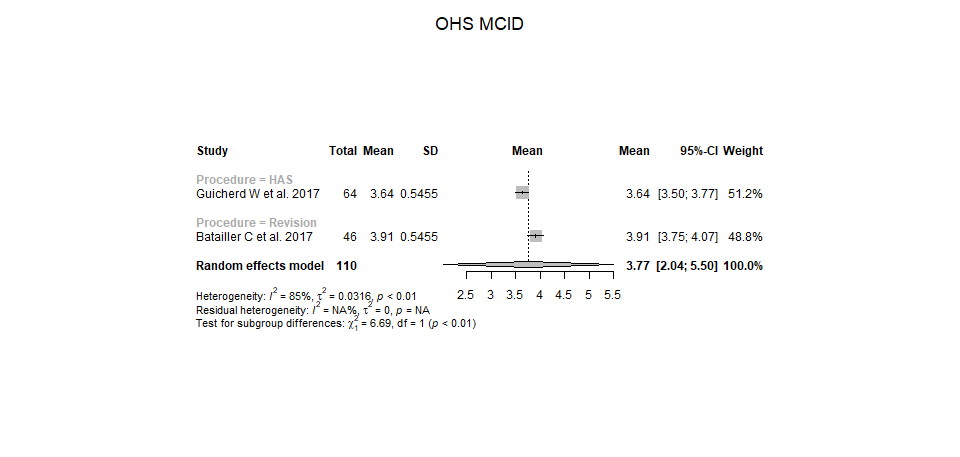

Supplement: Supplementary file 9 — FIGURE S9. Forest plot OHS MCID. [file OS-17-1899-s016.png]

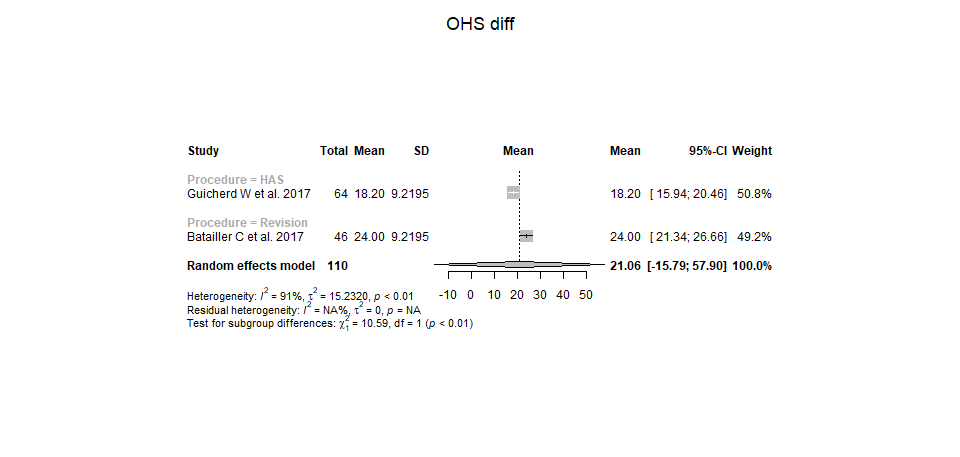

Supplement: Supplementary file 10 — FIGURE S10. Forest plot OHS difference. [file OS-17-1899-s017.png]

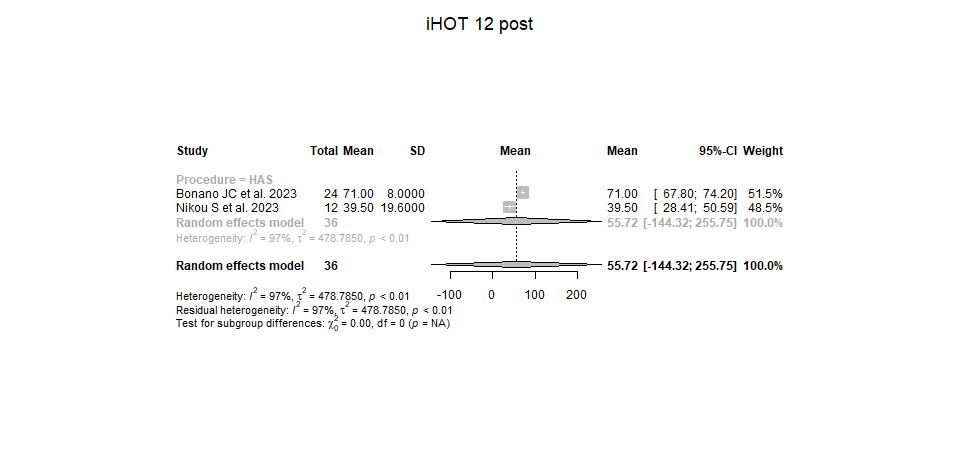

Supplement: Supplementary file 11 — FIGURE S11. Forest plot iHOT 12 postoperatively. [file OS-17-1899-s005.png]

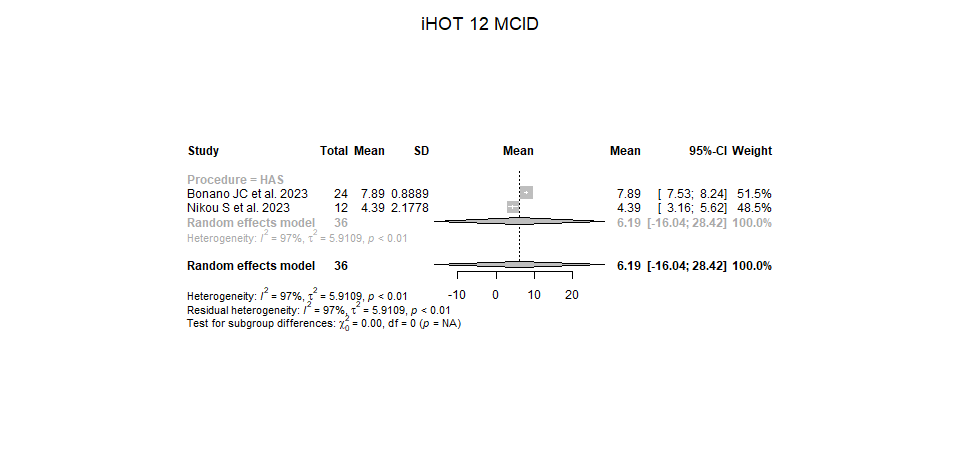

Supplement: Supplementary file 12 — FIGURE S12. Forest plot iHOT 12 MCID. [file OS-17-1899-s020.png]

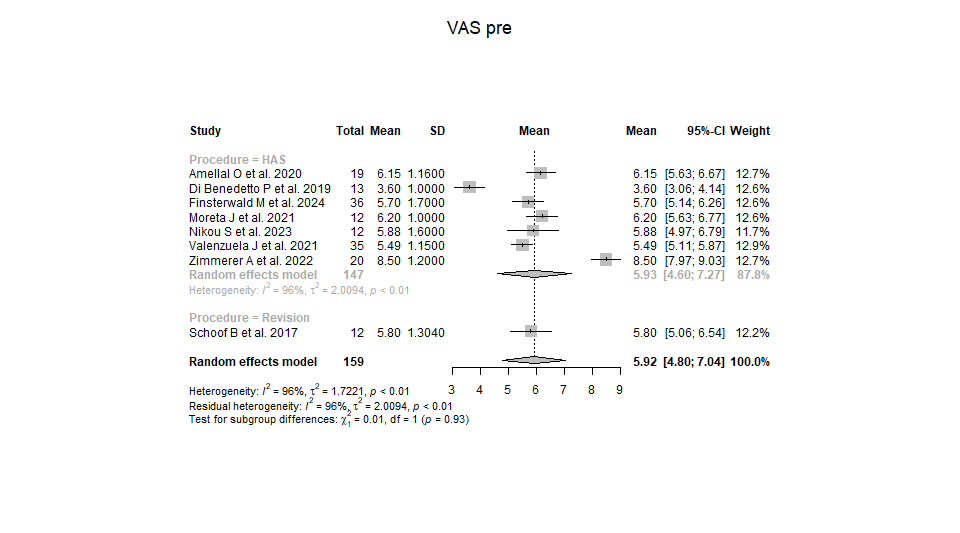

Supplement: Supplementary file 13 — FIGURE S13. Forest plot VAS preoperatively. [file OS-17-1899-s011.png]

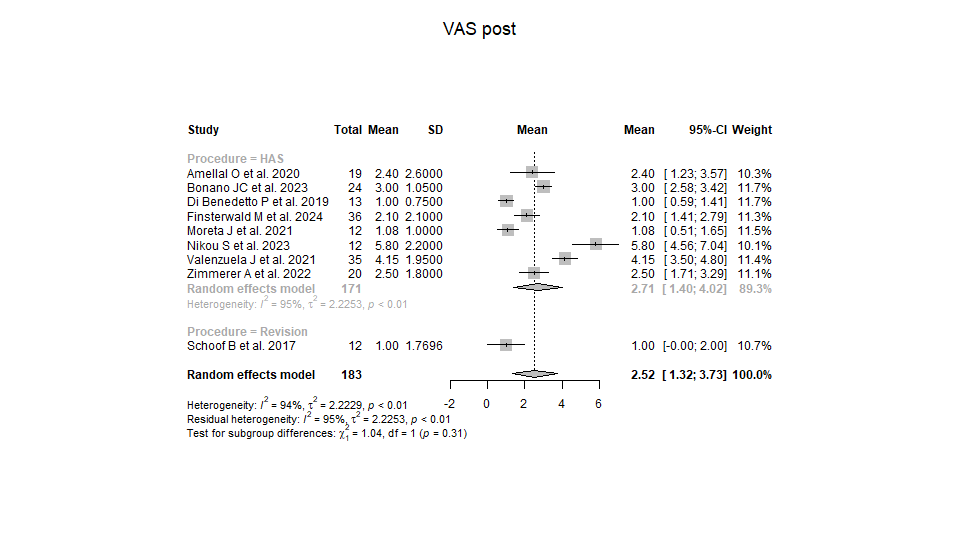

Supplement: Supplementary file 14 — FIGURE S14. Forest plot VAS postoperatively. [file OS-17-1899-s013.png]

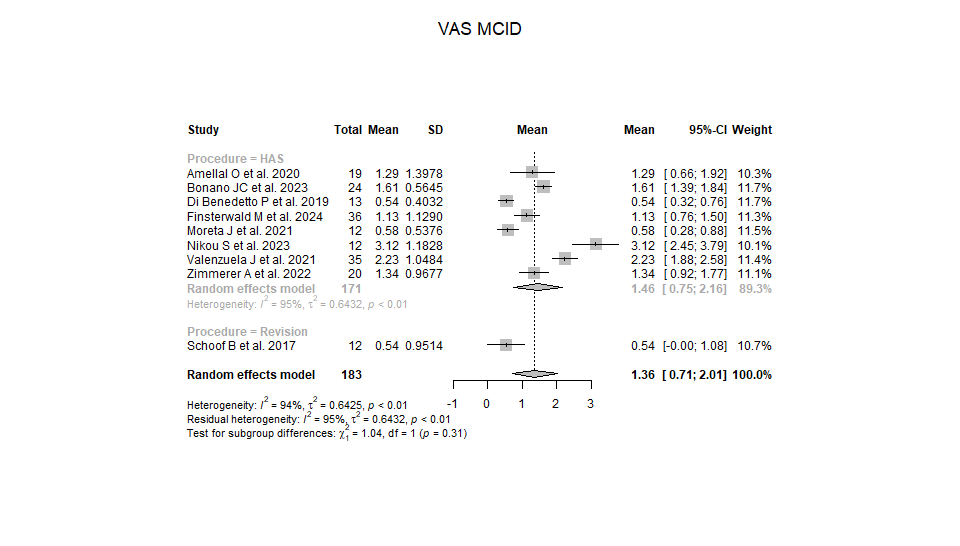

Supplement: Supplementary file 15 — FIGURE S15. Forest plot VAS MCID. [file OS-17-1899-s003.png]

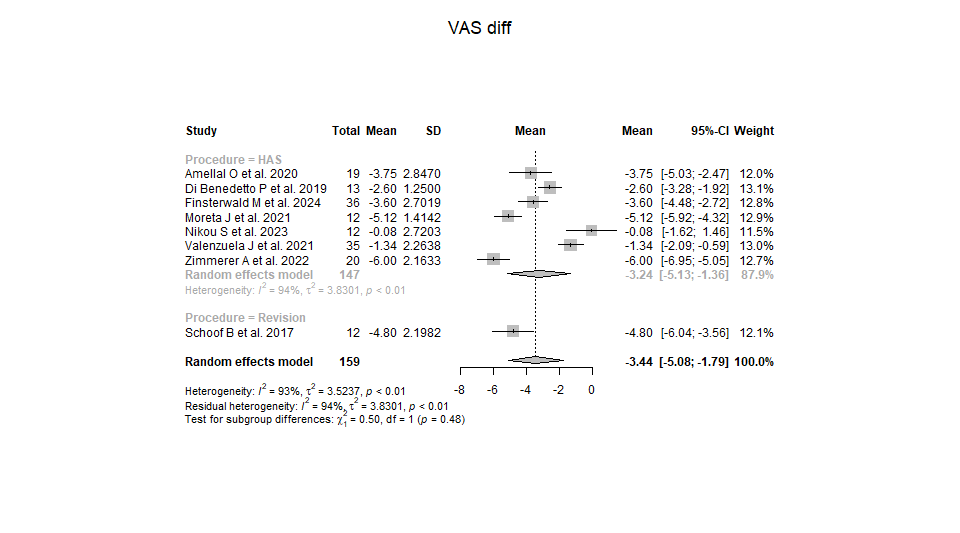

Supplement: Supplementary file 16 — FIGURE S16. Forest plot VAS difference. [file OS-17-1899-s008.png]

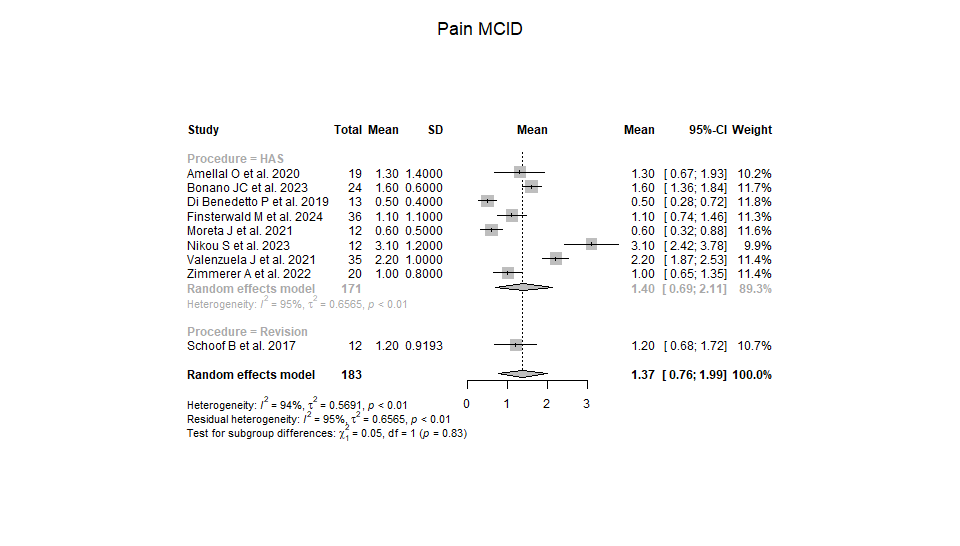

Supplement: Supplementary file 17 — FIGURE S17. Forest plot Pain MCID. [file OS-17-1899-s021.png]

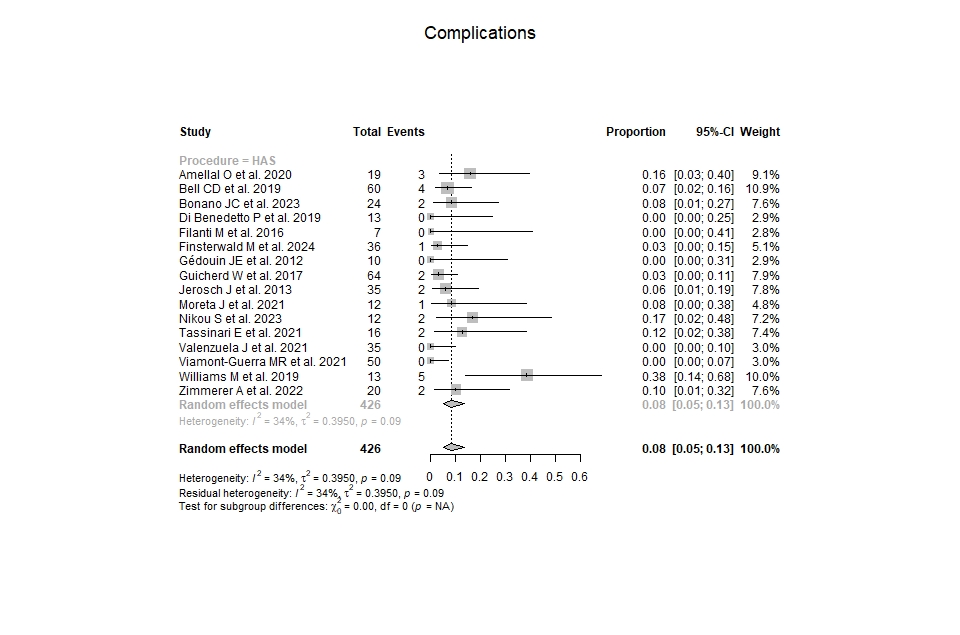

Supplement: Supplementary file 18 — FIGURE S18. Forest plot Complications. [file OS-17-1899-s004.png]

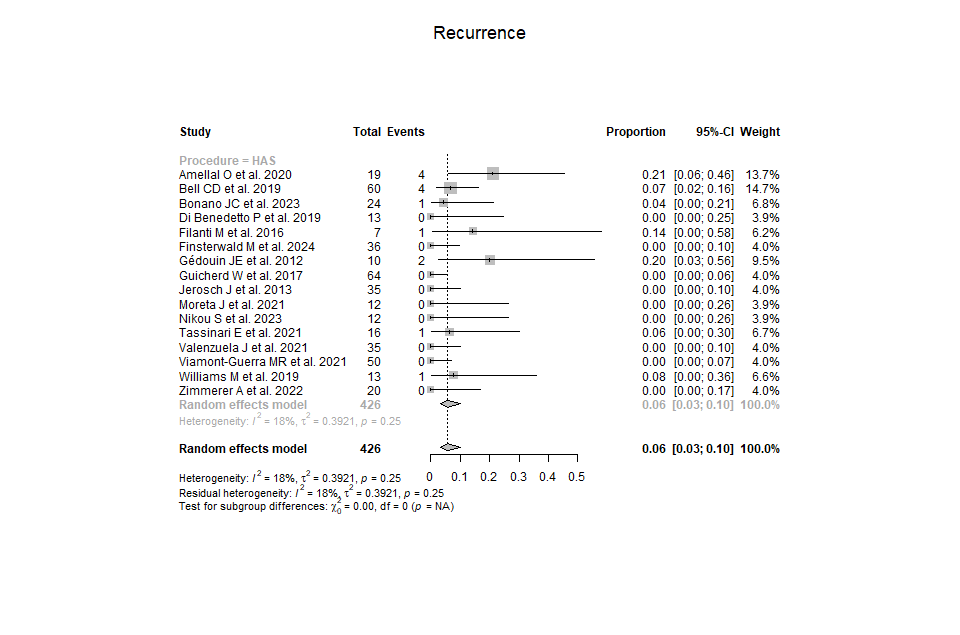

Supplement: Supplementary file 19 — FIGURE S19. Forest plot Recurrence. [file OS-17-1899-s012.png]

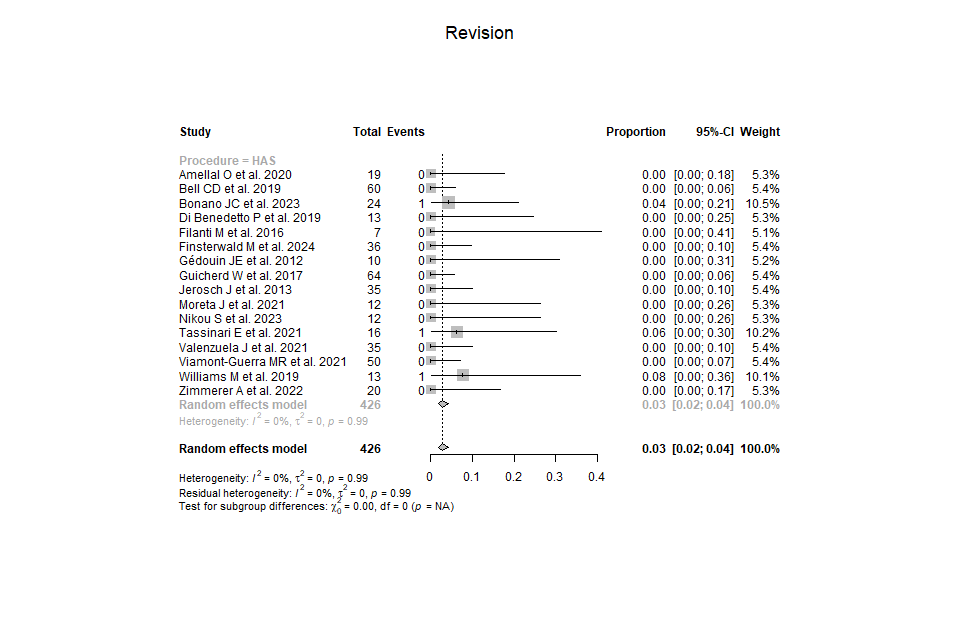

Supplement: Supplementary file 20 — FIGURE S20. Forest plot Revision. [file OS-17-1899-s007.png]
